# Preparation of ^18^O-labelled azaspiracids for accurate quantitation using liquid chromatography–mass spectrometry

**DOI:** 10.1007/s00216-023-04868-4

**Published:** 2023-08-02

**Authors:** Elliott J. Wright, Juris Meija, Pearse McCarron, Christopher O. Miles

**Affiliations:** 1 Biotoxin Metrology, National Research Council Canada, 1411 Oxford Street, Halifax, NS B3H 3Z1 Canada; 2 National Research Council, 1200 Montreal Road, Ottawa, ON K1A 0R6 Canada

**Keywords:** Azaspiracid, Matrix effect, Isotopic labelling, Oxygen-18, LC–MS, CRM-FDMT1, CRM-AZA-Mus

## Abstract

Azaspiracids (AZAs) are a group of polyether marine algal toxins known to accumulate in shellfish, posing a risk to human health and the seafood industry. Analysis of AZAs is typically performed using LC–MS, which can suffer from matrix effects that significantly impact the accuracy of measurement results. While the use of isotopic internal standards is an effective approach to correct for these effects, isotopically labelled standards for AZAs are not currently available. In this study, ^18^O-labelled AZA1, AZA2, and AZA3 were prepared by reaction with H_2_^18^O under acidic conditions, and the reaction kinetics and sites of incorporation were studied using LC–HRMS/MS aided by mathematical analysis of their isotope patterns. Analysis of the isotopic incorporation in AZA1 and AZA3 indicated the presence of four exchangeable oxygen atoms. Excessive isomerization occurred during preparation of ^18^O-labelled AZA2, suggesting a role for the 8-methyl group in the thermodynamic stability of AZAs. Neutralized mixtures of ^18^O-labelled AZA1 and AZA3 were found to maintain their isotopic and isomeric integrities when stored at −20 °C and were used to develop an isotope-dilution LC–MS method which was applied to reference materials of shellfish matrices containing AZAs, demonstrating high accuracy and excellent reproducibility. Preparation of isotopically labelled compounds using the isotopic exchange method, combined with the kinetic analysis, offers a feasible way to obtain isotopically labelled internal standards for a wide variety of biomolecules to support reliable quantitation.

**Graphical Abstract:**

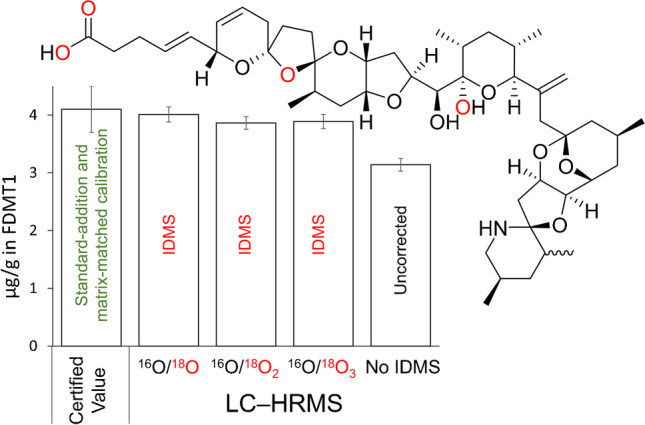

**Supplementary Information:**

The online version contains supplementary material available at 10.1007/s00216-023-04868-4.

## Introduction

**Fig. 1:**
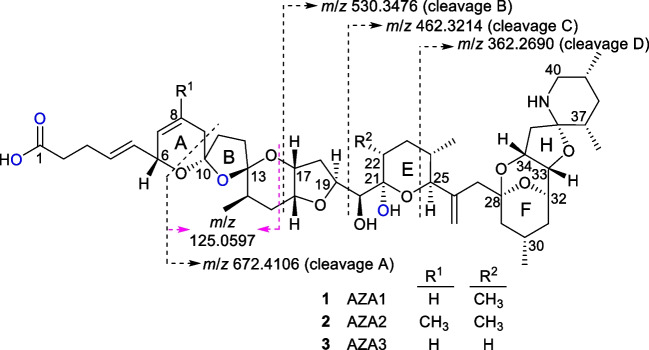
Structures of AZA1–3, showing the differences between the structures at C-8 and C-22, and characteristic mass spectral cleavages for unlabelled [M + H]^+^ of AZA1 in positive ionization mode. The four exchangeable oxygen atoms are shown in blue. The *m*/*z* values for cleavages A–C include neutral loss of water between C-21 and C-22, and cleavage D is believed to result from retro-Diels–Alder cleavage of the 21,22-dehydrated E-ring

Azaspiracids (AZAs) are a group of marine algal toxins that can accumulate in edible tissues of shellfish leading to human intoxication, characterized by nausea and gastrointestinal discomfort [1, 2]. To date, more than 50 AZA analogues have been reported [1–3], many of which have not had their structures unambiguously elucidated through NMR spectroscopy [2]. Of the well characterized AZAs, AZA1–3 (Fig. 1) are regulated in many jurisdictions, with monitoring typically performed by liquid chromatography–mass spectrometry (LC–MS) [4–6].

Matrix effects in LC–MS analysis are known to negatively affect the accuracy of measurement results, and isotope dilution is often employed to overcome these problems [7]. Presently, isotopically labelled AZAs are not available, and other methods must be used to mitigate matrix effects, such as standard-addition, matrix-matched calibration, dilution [3], or separating the AZAs from the matrix with selective binding methods such as boric acid gel [8] or immunoaffinity column chromatography [9, 10]. Using some of the aforementioned techniques, the National Research Council of Canada produced two mussel tissue matrix certified reference materials (CRMs) certified for AZA1–3 in the absence of available isotopically labelled standards, and these are available for use in assessing entire analytical methods for their accuracy [11–13].

Given the complex structure of most algal toxins, stable-isotope labelling of toxins has often been performed either biosynthetically through culturing in ^15^N-labelled media or by chemical reactions of the native toxin to incorporate isotopically labelled atoms [14–16]. AZAs are chemically unstable in acidic environments owing to isomerization and degradation [17, 18]. However, the reversible nature of acid-catalysed isomerization reactions presents an opportunity to incorporate isotopically labelled atoms such as ^18^O or ^2^H into exchangeable sites. To benefit from this exchange, the rate of incorporation into the preserved structure needs to be faster than the rate of isomerization into other stereoisomers. Minimal labelling of the target analyte (e.g., one ^13^C in phenol), addition of which results in a blended isotopic envelope from the combination of the native analyte’s natural isotopes and the minimally-labelled analyte, can be used for calibration by applying multiple linear regression [19], an approach that has been applied for routine use [20, 21]. Another approach is to apply double isotope-dilution, where the concentration of the labelled species does not need to be accurately determined, and calibration curve non-linearity from residual unlabelled compound, or interfering isotopes present naturally in the sample, can be easily and accurately modelled using rational functions as described by Pagliano et al. [22, 23].

Here we report ^18^O-labelling of AZA1, AZA2, and AZA3 with H_2_^18^O in acidic conditions and evaluate the use of labelled AZA1 and AZA3 as isotopic internal standards for performing accurate quantitation of AZAs in mussel tissue reference materials (RMs) by LC–MS.

## Materials and methods

### Reagents

^18^O-labelled water (97% oxygen-18) was from Cambridge Isotope Laboratories (Tewksbury, MA, USA). Trifluoroacetic acid (TFA; Reagent Plus grade, 99%) and ammonium acetate (LCMS grade) were from Sigma-Aldrich (Oakville, ON, Canada). Ammonium hydroxide (Certified ACS Plus, 28–30%) and MeCN (Optima LC–MS grade) were from Fisher Scientific (Whitby, ON, Canada). Deionized water was obtained from a Milli-Q Reference A + water purification system (Millipore Corp., Billerica, MA, USA). The matrix materials CRM-FDMT1 [11] and CRM-AZA-Mus [24] and the calibration solutions CRM-AZA1-b [25], CRM-AZA3-b [26], RM-AZA123 [27], and CRM-AZA1 were from the National Research Council (Halifax, NS, Canada; https://nrc.ca/crm), The calibration solution RM-AZA1 and the matrix material RM-AZA-Mus were as described by Quilliam et al. [28], and a specimen of synthetic AZA3 containing residual C-1 alcohol analogue of AZA3 was available from earlier work [29].

### Kinetic study of ^18^O-labelling for AZA1–3

An aliquot of RM-AZA123 (100 µL) was evaporated under N_2_ at ambient temperature. MeCN (80 µL) was added, followed by H_2_^18^O (20 µL). An aliquot (20 µL) of the mixture was immediately removed to represent the unreacted sample, and TFA (1 µL) was added to the remaining sample to initiate the isotopic exchange. The reaction mixture and the unreacted sample were both placed in the cooled autosampler tray (10 °C) and analysed by LC–HRMS at various times from 0 (the unreacted mixture) to 77 h along with an injection of RM-AZA123 at each time point.

### AZA1 ^18^O-labelling (feasibility study)

A mixture of CRM-AZA1 and CRM-AZA1-b in MeOH (approx. 1 µg AZA1) was dried under N_2_ at room temperature, reconstituted with 160 µL MeCN, and 40 µL of H_2_^18^O was added. Then, 2 µL of TFA was added to begin the reaction, with reaction progress monitored by LC–MS/MS. After 20 h of reaction, 170 µL of the solution was neutralized with ammonium hydroxide (0.15 M; 510 µL) and loaded onto an Oasis HLB (60 mg, 3 cc; Waters, Milford, MA, USA) solid-phase extraction (SPE) cartridge that had been preconditioned with 3 mL each of MeOH and 0.15 M ammonium hydroxide. The cartridge was washed with water until the eluent tested pH-neutral, aspirated under vacuum to dryness, and eluted with 250 µL MeCN. This eluent was used as an isotopically labelled spike stock solution, spiking 75 µL into aliquots (123 µL) of CRM-FDMT1 extract, MeOH, and four levels of a CRM-AZA1-b standard (6–170 ng/mL) in MeOH. Calibration standards were injected in triplicate and samples analysed with duplicate injections by LC–HRMS. The overlap between the isotopic patterns of the natural and ^18^O-labelled AZAs leads to nonlinear calibration curves which were modelled using a rational calibration function, *y* = (*a* + *bx*)/(1 + *cx*), as discussed by Pagliano et al. [23]. Fitting was done in Excel using ordinary least squares (supplementary Microsoft Excel spreadsheets in Pagliano et al. [23]). A small amount of unlabelled water (5 µL) was added to an aliquot (approx. 80 µL) of the MeOH spiked with ^18^O-labelled AZA spike stock to mimic the water that is inherently present in shellfish extracts and analysed by LC–HRMS after preparation, and again after 18 months of storage at −20 °C.

### AZA1 ^18^O-labelling (quantitation study)

Twenty ampoules of RM-AZA1 were combined and evaporated under nitrogen to give approx. 12 µg of AZA1, to which MeCN (640 µL) was added followed by H_2_^18^O (160 µL). TFA (8 µL) was added, the solution was mixed, and 50 µL was aliquoted into a separate vial for LC–HRMS analysis to monitor reaction progress. The reaction was maintained at 10 °C, and, after 48 h, the mixture was neutralized by adding 0.15 M ammonium hydroxide (3 mL). SPE was performed as described above, except that elution was performed sequentially with three portions of MeOH (500, 1000, and 2000 µL), and the fractions were analysed by LC–HRMS. An aliquot (370 µL) of the first fraction was added to MeOH (630 µL) for use as the ^18^O-labelled AZA1 stock solution for spiking extracts of the matrix materials. Materials were stored at −20 °C.

A mixed standard for AZA1 and AZA3 at 325.6 and 55.6 ng/mL, respectively, was prepared by combining aliquots of CRM-AZA1-b and CRM-AZA3-b gravimetrically in MeOH and diluted by serial additions of 200 µL to MeOH (400 µL) to generate a five-point standard curve. The ^18^O-labelled AZA1 stock (50 µL) was spiked into an aliquot of each level of mixed standard (200 µL), as well as into tissue reference material extracts and MeOH. Triplicate injections of all samples and standards were performed using both LC–MS/MS and LC–HRMS methods.

### Sample extractions

Extracts of CRM-FDMT1, CRM-AZA-Mus, and RM-AZA-Mus were prepared as described previously [13]. CRM-FDMT1 (0.35 g) was reconstituted with water (1.65 mL), vortex-mixed (30 s), and sonicated (60 s) to produce 2 g of wet-tissue-equivalent. Extractions were performed by adding MeOH (5.5 mL) to 2 g of wet tissue (or equivalent), vortex-mixed (2500 rpm, 3 min; model DVX-500, VWR Int., Radnor, PA, USA), and centrifuged (3950 g, 10 min; Sorvall Legend RT + , Thermo Scientific, Osterode, Germany). The supernatants were transferred to 25 mL volumetric flasks and the pellets subjected to three further identical extractions, with the combined supernatants made to volume in the flask.

### LC–MS/MS analysis

LC–MS/MS was performed on an Agilent 1260 LC system (Agilent Inc., Palo Alto, CA, USA) equipped with a binary pump, degasser, column oven (25 °C), and autosampler (6 °C). LC eluent was interfaced to an API4000 QTRAP mass spectrometer using a Turbospray ionization source (Sciex, Concord, ON, Canada), all controlled by Analyst 1.6.2 software. LC separations were on a Luna C18(2)HST column (50 × 2 mm, 2.7 µm; Phenomenex, Torrance, CA, USA) using gradient elution (0.3 mL/min) with a binary mobile phase of water (A) and 95% MeCN (B), each containing ammonium acetate (5 mM), from 15 to 100% B over 7 min, held at 100% B for 4 min, and re-equilibrated for 4 min. MS parameters were curtain gas 20 psi, capillary voltage 5.5 kV, temperature 450 °C, GS1/GS2 at 50 psi, CAD −3 arbitrary units, DP 70 V, EP 10 V, CE 70 eV, CXP 15 V. Analysis was by selected reaction monitoring (SRM) with 100 ms dwell times for each transition with Q1 ions selected at unit resolution for *m*/*z* 842.5, 844.5, 846.5, and 848.5 for AZA1; *m*/*z* 828.5, 830.5, 832.5, and 834.5 for AZA3; and *m*/*z* 856.5 for AZA2, all with the same Q3 ions measured using the low-resolution setting at *m*/*z* 362.2.

### LC–HRMS analysis

LC–HRMS was performed on an Agilent 1200 LC system equipped with a binary pump, degasser, column oven (25 °C), and autosampler (6 °C, except 10 °C for kinetic trials), connected to a Thermo Q Exactive HF mass spectrometer with a HESI source. HESI settings were spray voltage 3.0 or −2.7 kV, 350 °C capillary temperature, sheath and auxiliary gas at 35 and 10 arbitrary units, respectively, probe heater temperature 300 °C, and S-lens RF level set to 70. Full-scan analysis was performed at the 240,000 resolution setting in positive mode with an AGC target of 1 × 10^6^, a maximum C-trap fill time of 100 ms, and a scan range of 750–1100 *m*/*z*. Parallel reaction monitoring (PRM) settings included a default charge state of one, with an inclusion list containing the exact *m*/z values for incorporation of 0, 1, 2, 3, and 4 ^18^O atoms into [M + H]^+^ of AZA1 (842.5049, 844.5091, 846.5134, 848.5176, 850.5219), AZA2 (856.5206, 858.5248, 860.5290, 862.5333, 864.5375), and AZA3 (828.4893, 830.4935, 832.4977, 834.5020, 836.5062). MS/MS spectra were collected at the 15,000 resolution setting with an AGC target 2 × 10^5^, a maximum C-trap fill time of 50 ms, loop count of 5, isolation window of 0.7 *m*/*z*, and a stepped collision energy of 35, 70, and 90 eV. Extracted ion chromatograms and peak areas were obtained using a mass tolerance of ±5 ppm.

LC–HRMS data for kinetic analyses of isotope incorporation were obtained using a Synergi Max RP C_12_ column (50 × 2 mm, 3 µm; Phenomenex, Torrance, CA, USA) and detection by full-scan with PRM. The LC separation used the same gradient described above with flow was diverted to waste for the first 2 min and during column equilibration.

IDMS quantitation used the same Luna LC column and mobile phases as for LC–MS/MS analysis, with full-scan detection at the 120,000 resolution setting using polarity switching with an AGC target 1 × 10^6^, a maximum C-trap fill time of 100 ms and 200 ms positive and negative ionization modes, respectively, and a scan range of 750–900 *m*/*z*.

### Isotopic incorporation analysis

The NRC Isotopic Enrichment Calculator [30] was modified to accommodate multiple independent pools of isotopic enrichment per molecule, as well as the ability to model the isotopic incorporation of ^2^H and ^18^O (in addition to ^15^N and ^13^C) (https://metrology.shinyapps.io/isotopic-enrichment-calculator/). Data was processed by considering three pools of exchangeable oxygen sites, with two of the pools each having single-atom occupancy and the third pool having two-atom occupancy. A kinetic model involving 16 differential equations describing the 64 pseudo-first-order reactions of ^18^O-exchange among the 16 distinct isotopologues of the AZAs was applied to this data (Schemes S1 and S2). The rate constants and half-lives for the approach of the ^18^O-incorporation to equilibrium were obtained directly for each pool by fitting 3-parameter exponential curves to the data using SigmaPlot version 14.5 (Systat Software Inc., San Jose, CA, USA).

## Results and Discussion

### Isotope incorporation kinetics

An initial study was performed on a mixed standard of AZA1–3 to determine the feasibility of acid-catalysed incorporation of ^18^O from labelled water. Of particular interest was the rate of isomerization and other degradation reactions that AZAs are known to undergo [18, 31] relative to any isotopic incorporation. Reactions were monitored by LC–HRMS to assess the isotopomer composition of the [M + H]^+^ ions of AZA1–3 as well as the production of AZA isomerization products and their isotopomer compositions. Injections of RM-AZA123 at each kinetic time point were used to quantify changes in peak areas over the course of the kinetic trial to correct for drift in instrument sensitivity and/or sample evaporation. Kinetic data shown (Figures S1–4) is based on corrected peak areas, which were corrected according to the observed percentage change in response of the corresponding AZA in the control standard, to ensure that observed peak area changes were only due to the reaction.

### AZA1

**Fig. 2:**
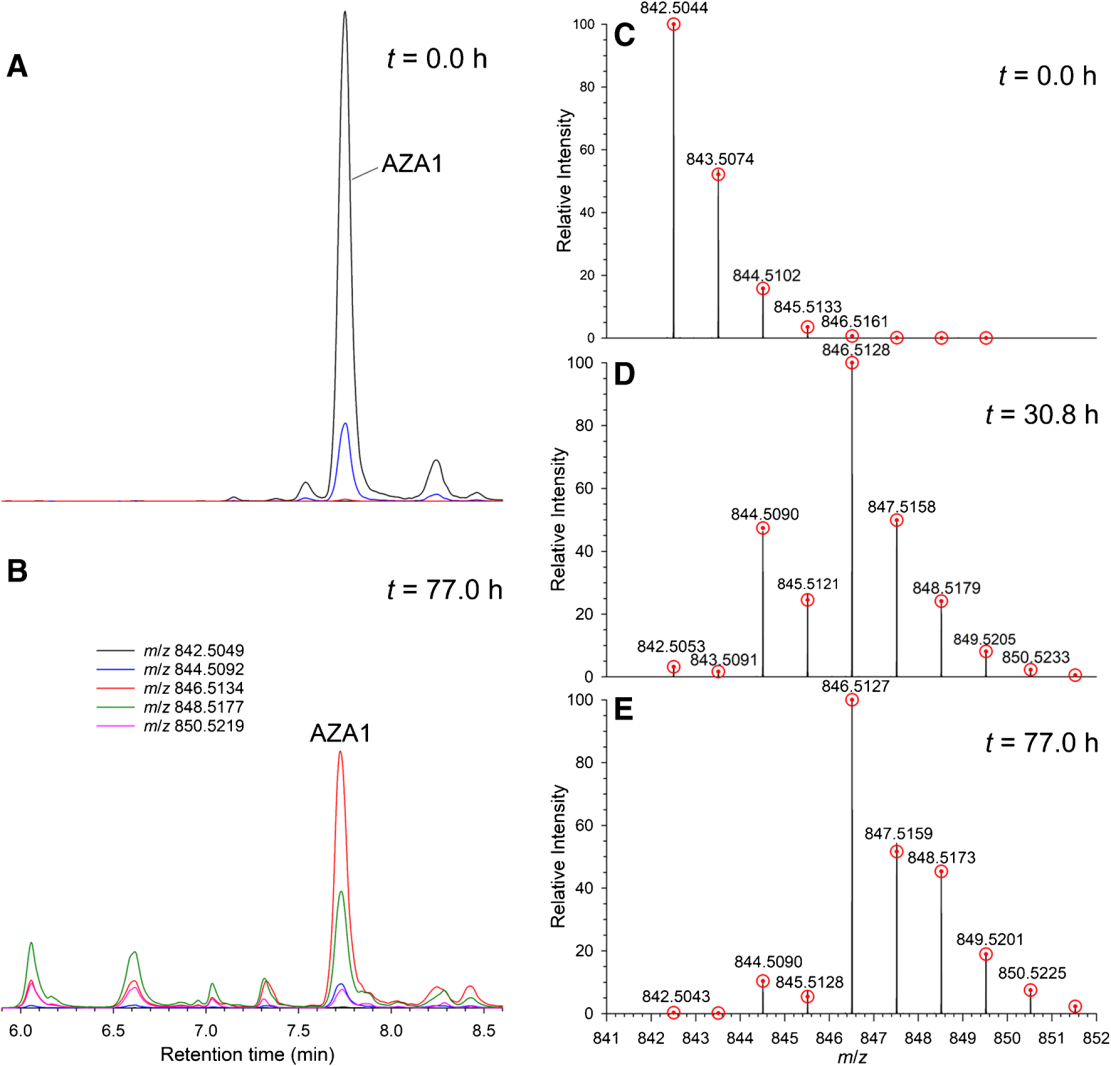
On the left are LC–HRMS full-scan chromatograms of AZA1 from the kinetic study extracted at the *m*/*z* values indicated in the legend, showing the distribution of isotopologues and isomers: **A** prior to starting the reaction and **B** after 77 h. Both chromatograms are shown with the same intensity scale. On the right are full-scan mass spectra obtained from the AZA1 peak showing the isotope distributions in [M + H]^+^: **C** prior to starting the reaction; **D** after 30.8 h of reaction (approx. 2.0% unlabelled AZA1, 30.0% [^18^O]AZA1, 60.2% [^18^O_2_]AZA1, 7.4% [^18^O_3_]AZA1), and 0.3% [^18^O_4_]AZA1, and **E** after 77.0 h of reaction (approx. 0.2% unlabelled AZA1, 7.1% [^18^O]AZA1, 68.3% [^18^O_2_]AZA1, 22.3% [^18^O_3_]AZA1, and 2.0% [^18^O_4_]AZA1). The red circles show fitted values from the NRC Isotopic Enrichment Calculator used to generate the isotopic incorporation data plotted in Fig. 3

**Fig. 3:**
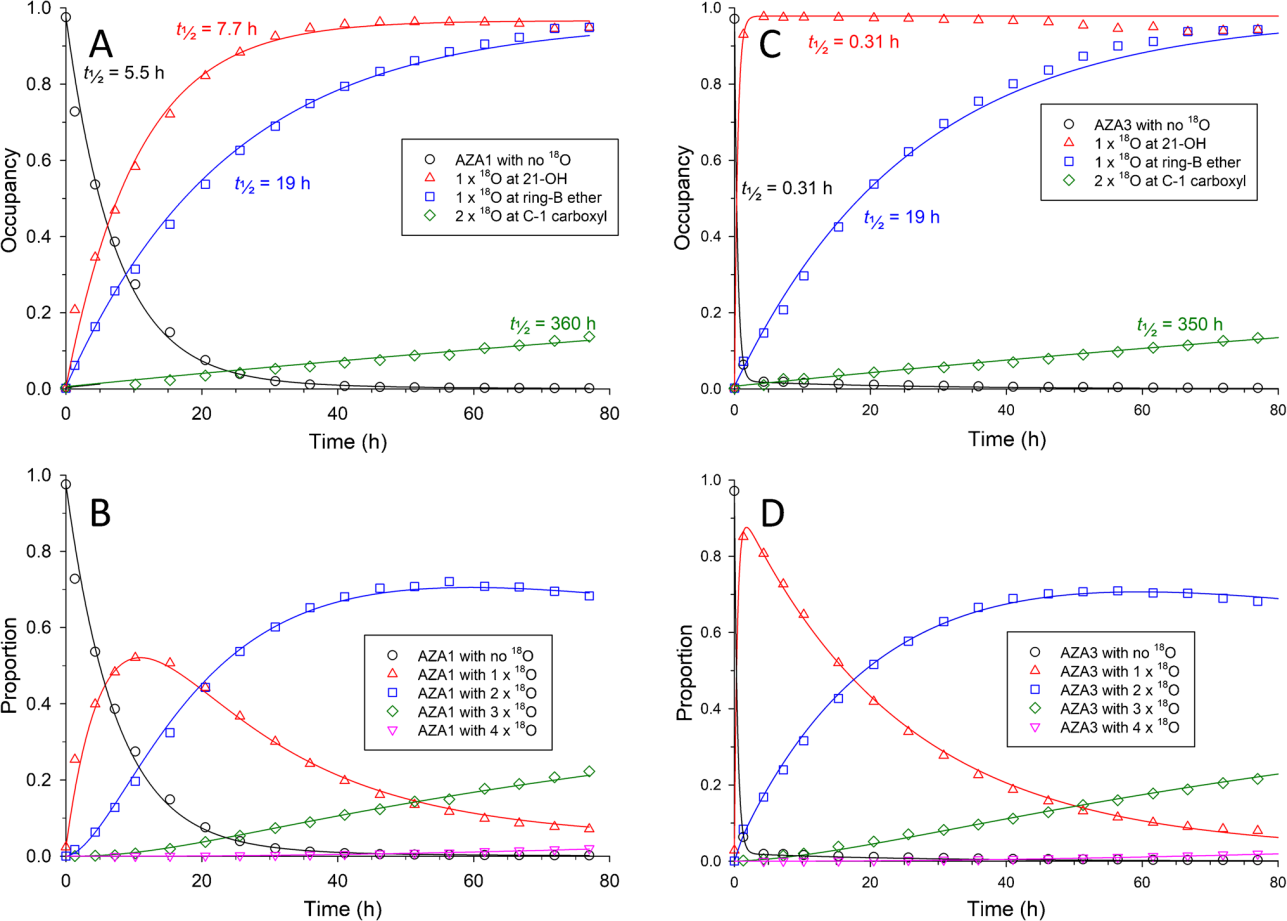
**A** Acid-catalysed incorporation of ^18^O into AZA1 based on analysis of the isotope envelope for [M + H]^+^ of AZA1 in full-scan spectra obtained during LC–HRMS analysis in the kinetic study. Mass spectral data was processed with the NRC Isotopic Enrichment Calculator (Fig. 2) with three isotopic pools. Locations of the isotopic pools on the molecule are inferred from LC–HRMS/MS data and chemical considerations. **B** The proportion of AZA1 molecules containing 0–4 ^18^O atoms with time, calculated using the NRC Isotopic Enrichment Calculator with 3 isotopic pools from data used to determine the incorporation kinetics. **C** and **D** show the corresponding data for AZA3. Lines were obtained from fitting a kinetic model involving 16 differential equations (Schemes S1 and S2) to the experimental data, but the indicated half-lives are for the approach to equilibrium as measured from fitting the data to 3-parameter exponential curves

Preliminary examination of the LC–HRMS data for the labelling reaction of AZA1 was performed by extracting for the calculated *m*/*z* of [M + H]^+^ ions for incorporation of 0–4 atoms of ^18^O (Fig. 2). Plotting their peak areas (Figure S1) versus time showed clear indications of significant incorporation of ^18^O into AZA1. LC–HRMS also showed the formation of several labelled isomers of AZA1, mostly at earlier retention times (Figs. 2B and S2). The isotopic profile of the [M + H]^+^ ion of AZA1 was monitored over the course of the reaction, using only the central part of the peak to minimize potential contributions from any partially co-eluting isomers of AZA1. The *m*/*z* of [M + H]^+^ for isomers of AZA2 and AZA3 and their ^18^O-isotopomers were sufficiently different from those of AZA1 that they did not interfere with this analysis. Examples of such spectra taken after 30.8 (Fig. 2D) and 77.0 h (Fig. 2E) of reaction illustrate the changes observed in the isotopic pattern of AZA1 over the course of the reaction, and clearly show rapid incorporation of two atoms of ^18^O, together with a slower incorporation of additional ^18^O. Due to significant contributions from ^13^C and other naturally occurring stable isotopes to the isotopomer envelope of AZA1 (Fig. 2C), the incorporation of ^18^O into AZA1 cannot be accurately determined directly from the chromatographic peak areas of its extracted ion [M + H + 2*n*]^+^ LC–HRMS peaks. We therefore modified the NRC Isotopic Enrichment Calculator, originally developed for uniform ^15^N-labelling of microcystins [14, 30], so that it could accommodate a wider range of isotopes being incorporated in up to four separate chemically equivalent pools. Results from application of this programme to the isotopic profiles from the LC–HRMS data of AZA1 over the course of the incorporation reaction are plotted in Fig. 3 and were consistent with one rapidly incorporating atom of ^18^O (pool 1), a second somewhat slower incorporating atom (pool 2), and a pair of very slowly incorporating atoms (pool 3) of ^18^O. These were attributed to the 21-OH, B-ring ether oxygen, and C-1 carboxyl oxygen atoms (Fig. 1), respectively, based on analyses of LC–HRMS/MS spectra and comparisons with kinetic data from AZA3 (Fig. 3), as discussed below. The sum of the intensities of the AZA1 isotopomers declined by approx. 20% over the course of the reaction (Figure S1), consistent with the partial conversion of AZA1 to isomeric products such as those visible in Fig. 2B.

### AZA2

AZA2 was much less stable in the acidic conditions used than AZA1 and AZA3 (Figure S2). The monoisotopic *m*/*z* for [M + H]^+^ of AZA2 decreased exponentially with a half-life of about 5 h, but the sum of all the isotopomer ions also decreased exponentially (half-life about 10 h), so that the final concentration of AZA2 at 77 h was just a few percent of the original value (Figures S2, S3). For many of the time points, analysis of the AZA2 mass spectra with the Isotopic Enrichment Calculator did not converge, or converged poorly with much higher *R*^2^ values than were observed when fitting data for AZA1 and AZA3, possibly due to chromatographic overlap with AZA2-isomers with differing isotopomer profiles. These kinetic results provide insight into the origins of the instability of AZAs and are discussed below in the section on identification of the sites of ^18^O-labelling of the AZAs.

### AZA3

Incorporation of ^18^O into AZA3 (Figs. 3 and S4) followed a nearly identical pattern to that found for AZA1 (Fig. 3), except that incorporation of the first atom of ^18^O occurred about 25-fold faster than for AZA1. Consequently, after only 1.3 h of reaction, about 85% of the AZA3 molecules were mono-labelled with ^18^O (with only 6% unlabelled) (Fig. 3), compared to only 25% of the AZA1 molecules (Fig. 3). The remaining ^18^O-incorporations into AZA3 occurred at rates that were identical (within experimental error) to those for AZA1. Similar levels of isomer formation were observed for AZA3 (Figure S2) as for AZA1, and these isomers did not interfere with analysis of the isotope incorporation into AZA3.

### Location of ^18^O-incorporation

Oxygen atoms in carbonyl groups and their derivatives, such as ketals and carboxylic acids, can often be exchanged using acid-catalysed exchange with water [32]. AZA1–3 contain four ketals (at C-10, C-13, C-21, and C-28) and one hemiaminal ether (at C-36) moiety, but the oxygen atoms in most of these are derived from intramolecular ring closure with neighbouring alcohol or amino groups (at C-6, C-17, C-25, C-32, C-33, C-34, and C-40), and aliphatic alcohols are not expected to exchange ^18^O under weakly acidic conditions [32]. Consequently, these AZAs contain three potential sites for incorporation of up to four atoms of ^18^O via acid-catalysed exchange with H_2_^18^O (Figure S5). Two of these sites are ketal oxygens, comprising the hemiketal OH at C-21 and the ether linkage in ring-B, whereas the other site is the two chemically equivalent oxygen atoms of the carboxylic acid group at C-1 (Fig. 1).

Comparison of the rate profiles for the three sites of incorporation in AZA1 and AZA3 (Fig. 3) showed that the two sites that exchanged more slowly were each labelled at essentially identical rates in both compounds, whereas the most rapidly labelled site exchanged about 25 times faster in AZA3 than in AZA1. The only difference in structure between AZA1 and AZA3 is the presence of a 22*R*-methyl group in the E-ring of AZA1 (Fig. 1). This strongly suggested that the rapidly exchanging site was the 21-OH group in the hemiketal and would be consistent with the reported [17] much greater propensity of AZA3 to undergo 21,22-dehydration, compared to AZA1. Consistent with this were the LC–HRMS/MS spectra of unlabelled AZA3 and [^18^O]AZA3 at 1.3 h of reaction time, when the first site of ^18^O-exchange was almost completely (approx. 93%) labelled while the remaining two sites were nearly unlabelled (7% and not detectable, respectively). The two spectra were essentially identical in every respect (Figures S6–S8), except that the first neutral loss from [M + H]^+^ of the mono-labelled AZA3 was 20.0143 Da (H_2_^18^O) and was followed by two losses of H_2_^16^O (Figure S7), indicating that it is the most easily lost water that carries the isotopic label. Similarly, the retro-Diels–Alder cleavage fragment at *m*/*z* 658.3938 (Fig. 1, cleavage A) of the mono-labelled AZA3, which also includes loss of one water molecule (from the E-ring), did not display any signs of the presence of ^18^O (Figure S7). Confirmation was obtained from examination of the isotopic profile of the in-source water loss in the LC–HRMS spectrum of the ^18^O-labelled AZA3 at 1.3 h (93% and 7% incorporation at two sites), which showed almost complete loss of the label (Figure S9) so that the ^18^O-content of the dehydration product was only 5%. At the same time, the 21,22-dehydroAZA3 (AZA25) that had formed in the reaction mixture contained only approx. 3% ^18^O and did not show detectable in-source loss of label (Figure S9), demonstrating that the site of rapid ^18^O-incorporation was at C-21.

A similar analysis of the LC–HRMS/MS spectra of mono-labelled [^18^O]AZA1 was complicated by the two fastest sites of incorporation differing in their exchange rates by less than threefold, so that at all the early time-points there was a mixture of AZA1 molecules labelled at one or the other position. Nevertheless, the labelling kinetics of AZA1 and AZA3, together with the HRMS/MS data from mono-labelled [^18^O]AZA1, were consistent with the site showing rapid incorporation of ^18^O in AZA1 as being the 21-OH hemiketal oxygen.

Examination of the LC–HRMS/MS spectrum of [M + H]^+^ of doubly labelled AZA1 at 77 h reaction time (Figures S10–S12) showed initial loss of H_2_^18^O (from 21-^18^OH) followed by two losses of H_2_^16^O (Figure S11). Product ions from retro-Diels–Alder cleavage in ring-A (Fig. 1, cleavage A) contained one atom of ^18^O (*m*/*z* 674.4133, C_38_H_58_O_8_^18^ON^+^, Δ −2.3 ppm) (Figure S11), but the product ions at *m*/*z* 530.3476, 462.3214, and 362.2690 (Fig. 1, cleavages B–D) did not contain any isotopic label (Figure S10), indicating that the second ^18^O label was incorporated between C-10 and C-15 and was therefore associated with ring-B. Interestingly, the low-mass product ion at *m*/*z* 125.0595 (C_7_H_9_O_2_^+^, *m*/*z* 125.0597, Δ =  − 0.9 ppm) in AZA1 was shifted, to *m*/*z* 127.0637 (C_7_H_9_O^18^O^+^, Δ =  − 2.1 ppm) (Figure S12), suggesting that this ion arises mainly through the fragmentation shown in Fig. 1, although another product ion that did not incorporate ^18^O also contributed to the *m*/*z* 125.0597 ion’s intensity (Figure S12). Corresponding results were obtained from analysis of the doubly labelled AZA3 at 77 h reaction time (Figures S13 and S14). The *m*/*z* 125.0597 ion does not appear to involve C-1, C-3, C-8, C-21, C-22, or C-23, as this ion was observed in LC–HRMS/MS spectra of known AZAs and their metabolites [17, 33] as well as in a synthetic specimen of the C-1 alcohol analogue of AZA3 (Figure S15). The finding that ring-B is involved in ^18^O-exchange under weakly acidic conditions, presumably via opening of the ketals at C-10 and/or C-13, provides a possible explanation of the observed instability of AZA2 under these conditions. AZA1 and AZA2 differ only by the presence of an olefinic methyl group in ring-A (at C-8) of the latter (Fig. 1). The presence of this methyl group presumably affects the relative rates of the viable ring closure pathways after exchange of the ring-B oxygen (via ring-opening), such that the pathway necessary to re-establish the original structure and stereochemistry in the C-6–C-17 region of AZA2 is no longer thermodynamically favoured.

Initial analysis of the mass spectra of AZA1 and AZA3 using the Isotopic Enrichment Calculator indicated the slow incorporation of two additional atoms of ^18^O (Figs. 2, 3, S1 and S4). The best fit to the data was obtained when these two atoms were included as one doubly occupied pool in the Isotopic Enrichment Calculator, and it is this data that is plotted in Fig. 3. The rate of incorporation of ^18^O into this pool was nearly 20-fold slower than that of the second, slower exchanging, ketal oxygen (in ring-B) in both AZA1 and AZA3. That, together with the apparent double occupancy, and that the incorporation rate was unaffected by methylation at C-22 (Fig. 3), was consistent with this site being associated with exchange into the carboxylic acid moiety at C-1, which is the only remaining site in AZAs that is capable of undergoing oxygen exchange under the mild acidic conditions used in this study. This was supported by the LC–HRMS/MS spectra of the [M + H]^+^ ions of the [^18^O_3_]AZA1 and [^18^O_3_]AZA3, both of which showed retention of two atoms of ^18^O (one in ring-B and the other at C-1) from their pseudomolecular ion water-loss clusters after the initial neutral loss of H_2_^18^O at C-21, but retention of only one ^18^O atom (in ring-B) in the product ions from their ring-A retro-Diels–Alder cleavages (cleavage A) (Figures S16 and S17), indicating the presence of one ^18^O atom on C-1 to C-9.

### ^18^O-Labelled AZA stability

Successful use of the ^18^O-labelled AZAs for quantitation by IDMS, or metabolic studies, requires that the isotopic labels be stable over the course of the experiment, including during the LC–MS analysis. Because the ^18^O-labels were introduced via mild acid-catalysed exchange with ^18^O-labelled water, chromatographic analyses were performed using an eluent buffered with ammonium acetate, rather than one of the commonly used acidic mobile phases [3], to reduce the likelihood of on-column isotopic exchange. In a preliminary study, efforts to stop the labelling reaction using SPE were tested with the procedure described, but using water instead of the weakly basic solution, and the eluted product was monitored by LC–MS/MS for 63 h. During this time, the ^18^O-labelled AZA1 peaks were observed to slowly back-exchange (> 17%), presumably due to traces of unlabelled water and acid in the SPE eluent. However, neutralization of the reaction mixture with ammonium hydroxide prior to SPE prevented this back-exchange, resulting in a stable product. When the isotopically labelled stock solution was spiked into MeOH and water to mimic the solvent composition of the shellfish extracts prepared in this study, the isotopic profile of AZA1 and AZA3 remained unchanged after 18 m of storage at −20 °C (Figure S18).

### IDMS quantitation with ^18^O-labelled AZAs

The certified values and their associated uncertainties for each AZA in CRM-FDMT1 and CRM-AZA-Mus are the sums for each main AZA and its corresponding 37-epimer [11, 24]. Under acidic mobile-phase conditions, AZAs, and their corresponding 37-epimers typically co-elute, but the neutral mobile phase conditions used in this study resolved the main AZA peaks from their epimers.

### IDMS feasibility study

**Fig. 4:**
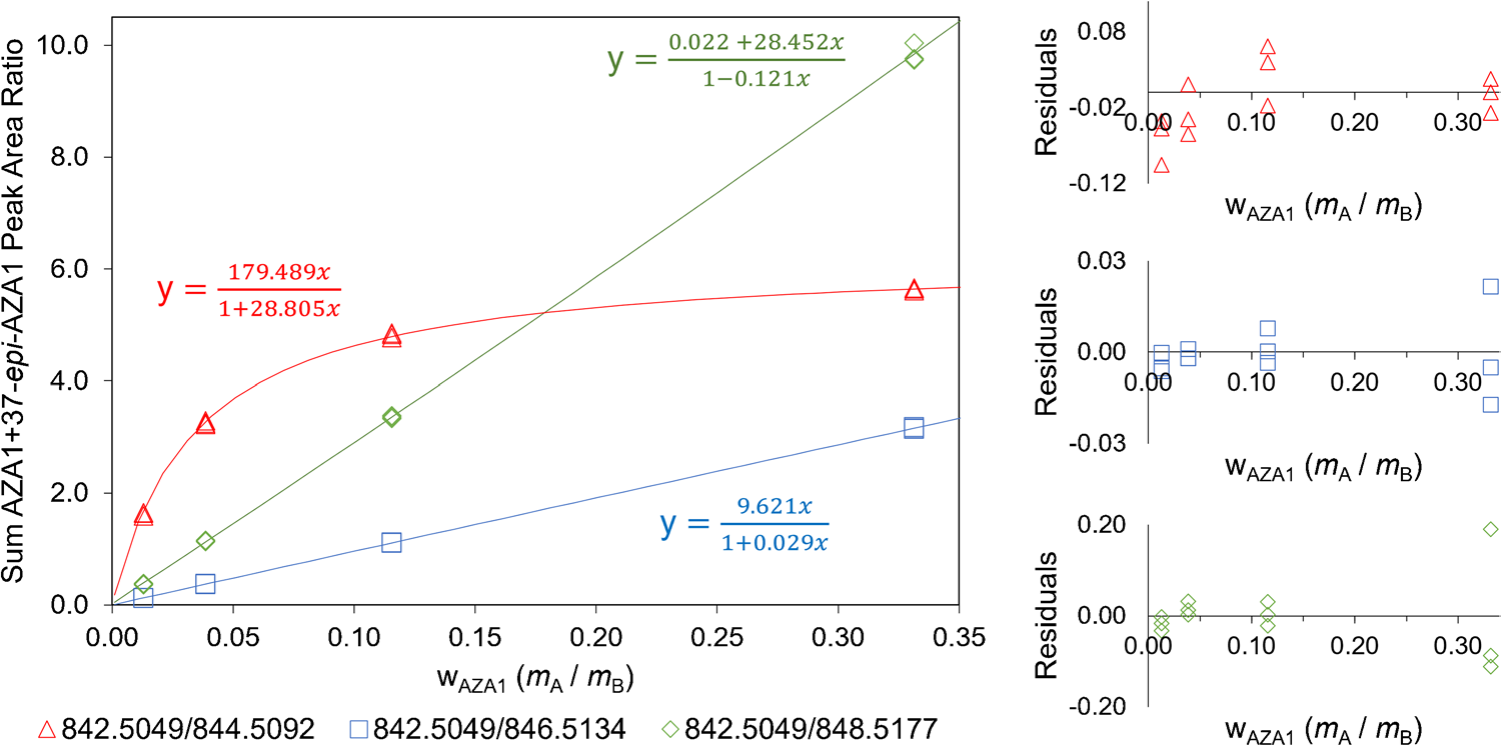
LC–HRMS IDMS calibration curves for AZA1 analysed in triplicate in the feasibility trial prepared from the peak area sum of AZA1 and 37-*epi*-AZA1 (native) divided by that of the singly (red triangles), doubly (green diamonds), or triply (blue squares) isotopically labelled AZA1 and 37-*epi*-AZA1, and applying the Padé[1,1] calibration model [23] using µg/g mass fraction of AZA1 in calibration standard corrected for dilution (mass of calibration standard (*m*_A_) and mass of isotopic spike solution (*m*_B_)). Residuals plots are shown (right) for each calibration curve

**Fig. 5:**
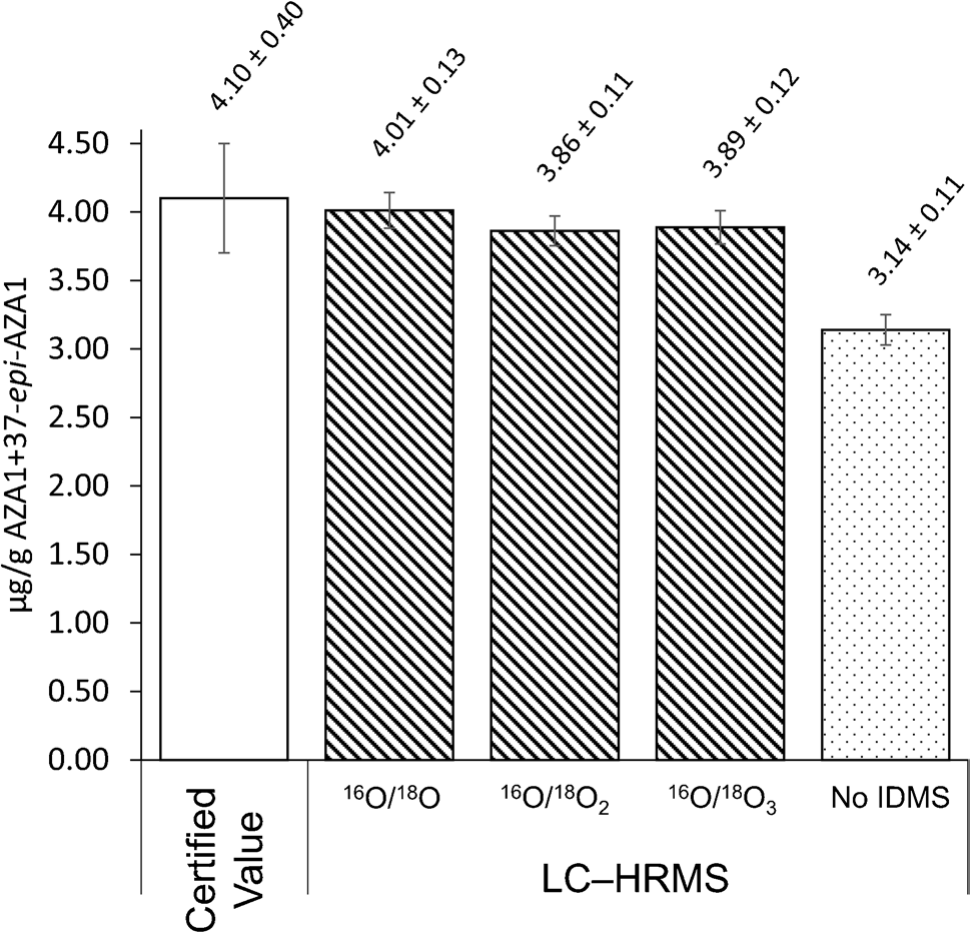
Quantitation results for the sum of AZA1 and 37-*epi*-AZA1 in CRM-FDMT1 from the feasibility study using LC–HRMS full-scan detection. The certified value is shown for CRM-FDMT1, with an error bar showing the stated uncertainty alongside results from isotope dilution using singly, doubly, or triply labelled AZA1 + 37-*epi*-AZA1 for calibration, and comparing to external calibration results without isotope dilution (No IDMS). Error bars are the propagated experimental uncertainty considering contributions from calibrant uncertainty, calibration model, replicate analysis, and sample preparation. Results were similar to those obtained later in the quantitation study (Table 1)

Having established the feasibility of labelling AZAs with ^18^O, the rate of incorporation at the exchangeable positions in the molecules, and the stability of the labels, labelled AZA1 was prepared in sufficient quantity for a feasibility trial to quantitate AZA1 in CRM-FDMT1 using IDMS. Progress of the labelling reaction was again monitored by LC–HRMS until the native AZA1 was nearly depleted, before neutralization and SPE. Single spikes of the resulting labelled stock solution into aliquots of the calibration standards and CRM-FDMT1 extract were analysed using LC–HRMS. For quantitation, the measured AZA1 was from a mixed signal of AZA1 in the sample extract and any residual native AZA1 in the isotopically-labelled spike. Likewise, the isotopomers measured were due to a mixture of the ^18^O-labelled AZA1 from the spike and the naturally occurring isotopomer peaks of unlabelled AZA1 from the sample. For this reason, non-linear calibration was required to accurately model the response ratio of the sum of the experimental peak areas for the natural AZA1 and its epimer divided by that of the corresponding isotopically labelled peaks against the mass fraction of the sum of AZA1 + 37-*epi*-AZA1 corrected for dilution with the isotopic spike solution (Fig. 4). The random distribution of residuals about the *x*-axis in the residuals’ plots (Fig. 4) demonstrated suitability of the calibration model, and concentrations determined using LC–HRMS with, and without, isotope dilution are shown in Fig. 5. Concentrations of AZA1 + 37-*epi*-AZA1 in CRM-FDMT1 obtained here using IDMS were all within the stated uncertainty of the certified value determined previously by standard-addition and matrix-matched calibration [11]. Given the results obtained, the feasibility of this method for quantitation warranted a more thorough investigation.

### IDMS quantitation study

To prepare sufficient quantities of labelled stock solution for a more comprehensive quantitative study, a larger amount of RM-AZA1 was used, with the reaction progress again followed by LC–HRMS. The reaction was neutralized by SPE after 48 h, and the ^18^O_2_-labelled AZA1 + 37-*epi*-AZA1 in the SPE fractions was estimated against an in-house standard (157 ng/mL) using a single-point calibration, and indicated approximately 0.5 µg in the first fraction, 1.3 µg in the second fraction and 0.3 µg in the third fraction. For quantitative analysis, the first elution fraction contained an adequate amount of material for further use. Additionally, given the presence of an AZA3 impurity in RM-AZA1 [28], the SPE fractions also contained low levels of labelled AZA3 (approx. tenfold lower than for labelled AZA1 in the first SPE fraction) that could also be used for IDMS quantitation of AZA3.

This isotopically labelled stock solution was used to spike calibration standards and extracts of CRM-AZA-Mus, CRM-FDMT1, and RM-AZA-Mus, and samples were analysed with both LC–HRMS and LC–MS/MS methods. Because the labelled AZAs did not separate chromatographically from native AZAs (e.g. Figure S19), IDMS with ^18^O-labelled AZAs is expected to correct for any potential matrix effects on quantitation. The results from the IDMS quantitation study are shown in Table 1. For AZA1, mean results ± standard deviation of the mean results for calibration with all three isotopic labels using both methods were 3.85 ±0.12 µg/g for CRM-FDMT1 and 1.03 ± 0.03 µg/g for CRM-AZA-Mus, in good agreement with the certified values for the two materials (Table 1). Mean results and standard deviation calculated for RM-AZA-Mus were 0.55 ±0.03 µg/g, higher than the non-certified values previously assigned (Table S1), possibly due to a combination of no correction for matrix effects, incomplete extraction recovery, and use of non-certified standards. AZAs were reported [12] to experience 16–24% suppression in CRM-AZA-Mus extracts analysed by SRM using similar LC–MS/MS conditions. For AZA1 in this study, matrix suppression was not this severe, although AZA3 concentrations determined without IDMS were clearly suppressed (13–20%) whereas IDMS methods yielded results in agreement with certified values (Table 1). Matrix effects were significant for AZA1 in the feasibility study (Fig. 5); however, the reproducibility of the IDMS results in the two separate experiments (< 2% difference between the feasibility (Fig. 5) and quantitation studies (Table 1)), and the accuracy of all the results compared to the CRM certified values demonstrates that quantitation of AZAs using IDMS was successful. Furthermore, the non-linear calibration methods used in this study demonstrate the ability to calibrate using a singly ^18^O-labelled AZA, meaning that calibration for AZA2 may also be feasible for reaction mixtures quenched prior to significant isomerization (e.g., before 10 h, see Figures S2 and S3). Given that accurate results were obtained using singly, doubly, and triply labelled AZAs, additional optimization (e.g. faster quenching) of the isotopic labelling reaction could result in a higher yield of usable spike, greatly increasing the practicality of such methods for routine use.

**Table 1:** Measured concentrations of AZA1 and AZA3 (µg/g) for CRM-FDMT1 and CRM-AZA-Mus, from the quantitation study. LC–MS-based values are mean results (uncertainty) using external calibration, or using IDMS with singly, doubly, or triply ^18^O-labelled AZA peaks from the spiked isotopically-labelled stock solution^*a*^

		CRM-AZA-Mus		CRM-FDMT1	
Method	Calibration	AZA1	AZA3	AZA1	AZA3
LC–HRMS	External calibrant	1.04 (0.04)	0.19 (0.01)	3.93 (0.22)	0.93 (0.04)
	IDMS 1 × ^18^O	1.00 (0.03)	0.24 (0.01)	3.77 (0.12)	1.05 (0.03)
	IDMS 2 × ^18^O	1.00 (0.03)	0.24 (0.01)	3.78 (0.11)	1.08 (0.06)
	IDMS 3 × ^18^O	1.01 (0.03)	0.23 (0.01)	3.85 (0.11)	1.04 (0.03)
LC–MS/MS	External calibrant	1.04 (0.03)	0.17 (0.00)	3.79 (0.12)	0.83 (0.02)
	IDMS 1 × ^18^O	1.06 (0.04)	0.24 (0.01)	4.09 (0.16)	1.08 (0.04)
	IDMS 2 × ^18^O	1.03 (0.03)	0.23 (0.01)	3.76 (0.11)	1.09 (0.04)
	IDMS 3 × ^18^O	1.05 (0.03)	0.23 (0.01)	3.85 (0.11)	1.07 (0.05)
Certified value (uncertainty)^*b*^		1.16 (0.10)	0.21 (0.02)	4.1 (0.40)	0.96 (0.10)

^*a*^AZA concentrations are reported as the sums of each AZA plus its 37-epimer. Similar values were obtained for AZA1 in FDMT1 in the feasibility study (Fig. 5). Results for the in-house reference material RM-AZA-Mus are shown in Table S1. Uncertainties given for IDMS results consider the contributions from calibrant uncertainty, calibration model, replicate analysis, and sample preparation. ^*b*^Certified values and their stated uncertainties for the CRMs [11, 24]

## Conclusions

Incorporation of ^18^O into AZAs was achieved and the locations of incorporation proposed with support from MS/MS and kinetic data. Notable differences in isomerization products and reaction rates were dependent on the presence of C-8 and C-22 methyl groups. Successful stabilization of the labelled AZAs and chromatographic separation of isomerization products from the native AZA compound allowed quantitative double isotope-dilution LC–MS experiments to be used without the need to quantitate the concentration of labelled species. Accurate quantitation of AZA1 and AZA3 was demonstrated in two matrix CRMs. Given the successful implementation of this method, future work to stabilize other ^18^O-labelled AZAs (e.g. AZA2) for quantitative use, or application to other similarly reactive compounds, is worthy of investigation. This work also demonstrated a procedure to produce labelled species for further purification that could be useful for other types of studies, such as metabolism and toxicology.

### Supplementary information

Below is the link to the electronic supplementary material.

Supplementary file1 (PDF 1.72 MB)

## Abbreviations

AZA: Azaspiracid
CE: Collision energy
CRM: Certified reference material
IDMS: Isotope-dilution mass spectrometry
LC–HRMS/MS: Liquid chromatography with high-resolution tandem mass spectrometry
LC–MS: LC with mass spectrometry
LC–MS/MS: LC with tandem mass spectrometry
PRM: Parallel reaction monitoring
RM: Reference material
SPE: Solid-phase extraction
SRM: Selected reaction monitoring
TFA: Trifluoroacetic acid
