# Supplementary material for: Preparation of 18O-labelled azaspiracids for accurate quantitation using liquid chromatography–mass spectrometry
Source: Anal Bioanal Chem. 2023 Aug 2;415(24):5973–83. doi: 10.1007/s00216-023-04868-4 (PMC10556123; doi:10.1007/s00216-023-04868-4)
Supplement: Supplementary file 1 — Supplementary file1 (PDF 1.72 MB) [file 216_2023_4868_MOESM1_ESM.pdf]

Preparation of ^{18}O-labelled azaspiracids for accurate quantitation using liquid chromatography–mass spectrometry

Elliott J. Wright, Juris Meija, Pearse McCarron, and Christopher O. Miles

Table of Contents

**Figure S1**	LC–HRMS peak intensities of [$^{18}O_n$]AZA1 (n = 0–4) with time	S2
**Figure S2**	LC–HRMS chromatograms showing isomerization of AZA1–3 with time	S3
**Figure S3**	LC–HRMS peak intensities of [$^{18}O_n$]AZA2 with time (n = 0–4)	S4
**Figure S4**	LC–HRMS peak intensities of [$^{18}O_n$]AZA3 with time (n = 0–4)	S5
**Figure S5**	Scheme showing the exchangeable and non-exchangeable oxygens in AZA1	S6
**Figure S6**	LC–HRMS/MS spectrum of [^{18}O]AZA3 at 1.3 h	S7
**Figure S7**	LC–HRMS/MS spectrum of [^{18}O]AZA3 at 1.3 h (expansion)	S8
**Figure S8**	LC–HRMS/MS spectrum of [^{18}O]AZA3 at 1.3 h (expansion)	S9
**Figure S9**	LC–HRMS and MS spectra of AZA3 and 21,22-dehydroAZA3 at 1.3 h	S10
**Figure S10**	LC–HRMS/MS spectrum of [$^{18}O_2$]AZA1 at 77 h	S11
**Figure S11**	LC–HRMS/MS spectrum of [$^{18}O_2$]AZA1 at 77 h (expansion)	S12
**Figure S12**	LC–HRMS/MS spectrum of [$^{18}O_2$]AZA1 at 77 h (expansion)	S13
**Figure S13**	LC–HRMS/MS spectrum of [$^{18}O_2$]AZA3 at 77 h	S14
**Figure S14**	LC–HRMS/MS spectrum of [$^{18}O_2$]AZA3 at 77 h (expansion)	S15
**Figure S15**	LC–HRMS/MS spectra of AZA3 and its C-1 alcohol analogue	S16
**Figure S16**	LC–HRMS/MS spectra of [^{18}O]AZA1 and [$^{18}O_2$]AZA1 at 77 h (expansion)	S17
**Figure S17**	LC–HRMS/MS spectra of [^{18}O]AZA3 and [$^{18}O_2$]AZA3 at 77 h (expansion)	S18
**Figure S18**	LC–HRMS spectra of ^{18}O-labeled AZA1 before and after storage	S19
**Figure S19**	LC–HRMS quantitation of AZA1 with and without isotope dilution	S20
**Scheme S1**	Schematic for kinetic model of ^{18}O-istopologue formation for AZAs	S21
**Scheme S2**	Mathematical model for kinetic analysis of ^{18}O-exchange, and results	S22
**Table S1**	IDMS quantitation of AZA1 and AZA3 in RM-AZA-Mus	S24
**Literature Cited**		S25

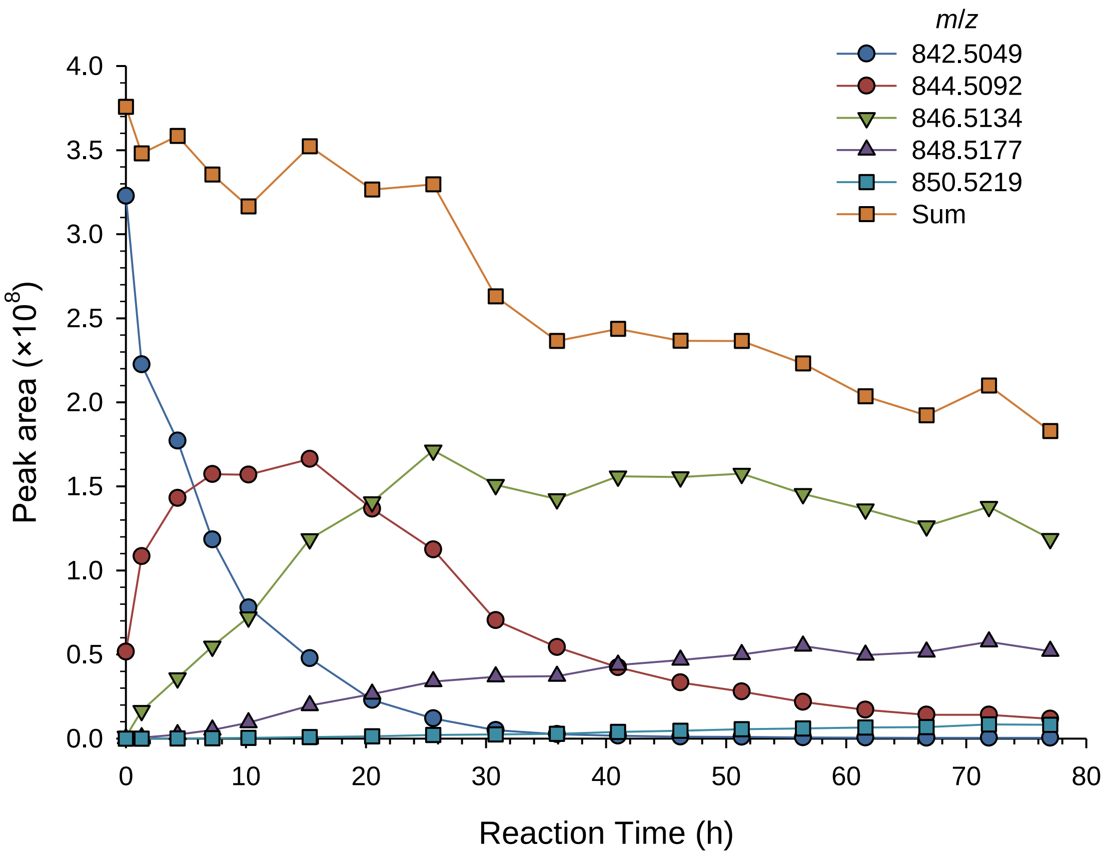

Figure S1. Acid-catalysed incorporation of 0–4 ^{18}O atoms into AZA1 based on analysis of LC–HRMS full-scan spectra for the ions listed (± 5 ppm), with the sum of all peak areas to demonstrate overall losses of AZA1. Note that this analytical approach does not correct for signal interference arising from isotopes naturally present in AZAs, such as ^{13}C.

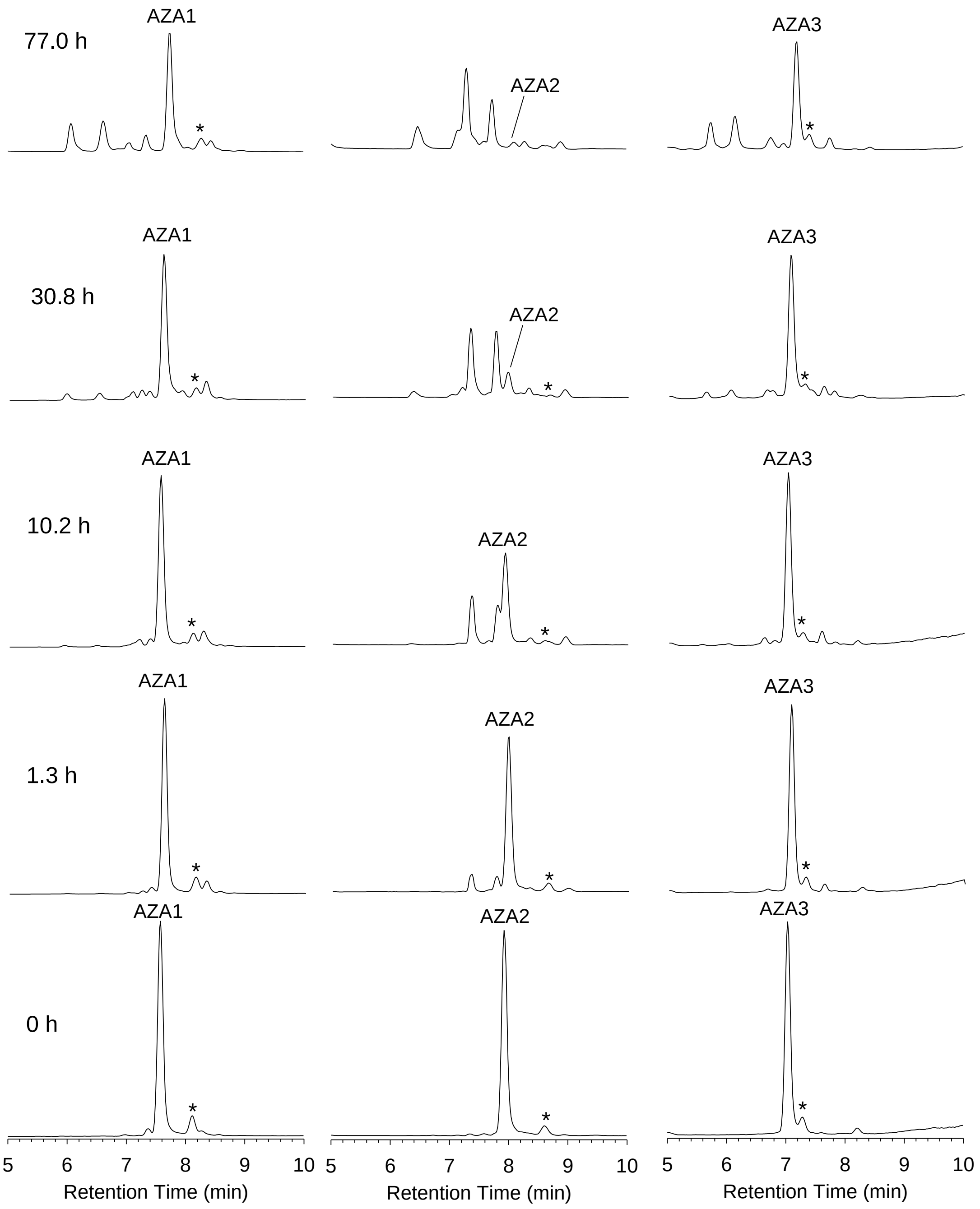

Figure S2. Selected LC–HRMS extracted ion chromatograms (Max RP method) for AZA1 (leftmost column, *m*/*z* range 842.4–850.6), AZA2 (middle column, *m*/*z* range 856.4–864.6) and AZA3 (rightmost column, *m*/*z* range 828.4–836.6) during the labelling reaction. The 37-*epi*-AZA1–3 peaks are marked with an "*" when present. Note: pH of mobile phase changed over the course of the run, causing retention times to slowly shift.

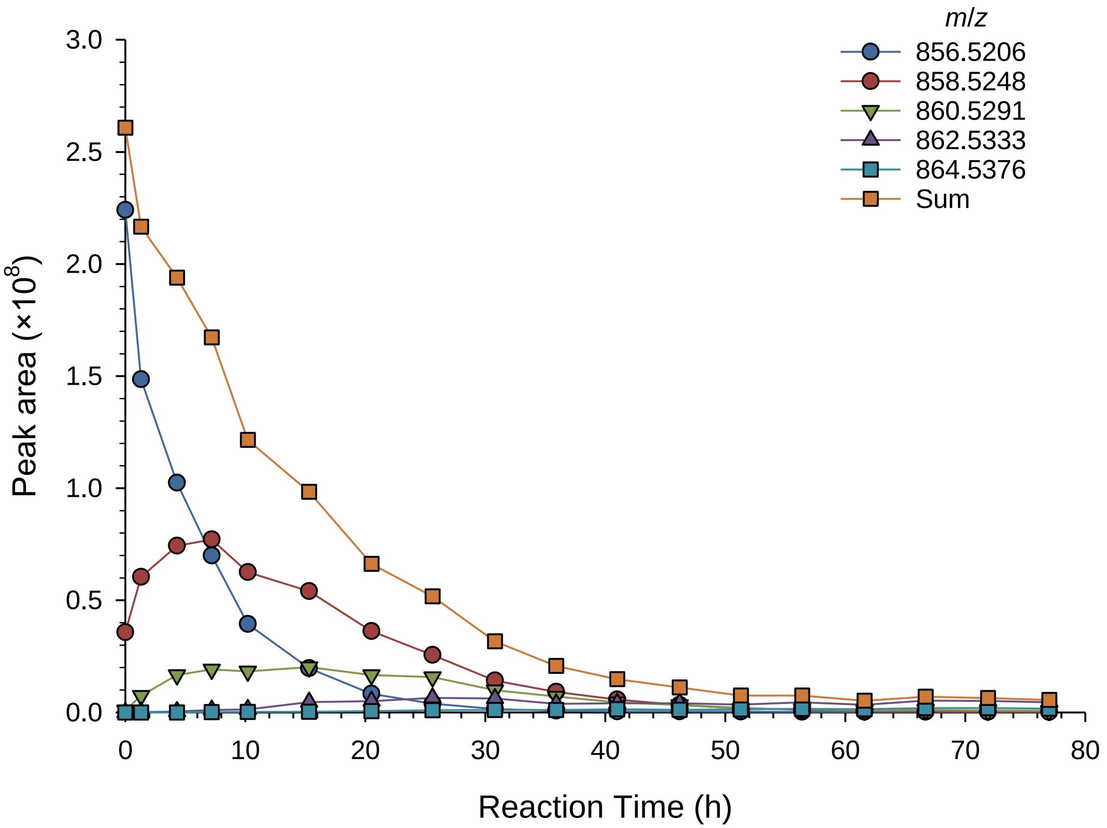

Figure S3. Acid-catalysed incorporation of 0–4 ^{18}O atoms into AZA2 based on analysis of LC–HRMS full-scan spectra for the ions listed (± 5 ppm), with the sum of all peak areas to demonstrate overall losses of AZA2. Note that this analytical approach does not correct for signal interference arising from isotopes naturally present in AZAs, such as ^{13}C.

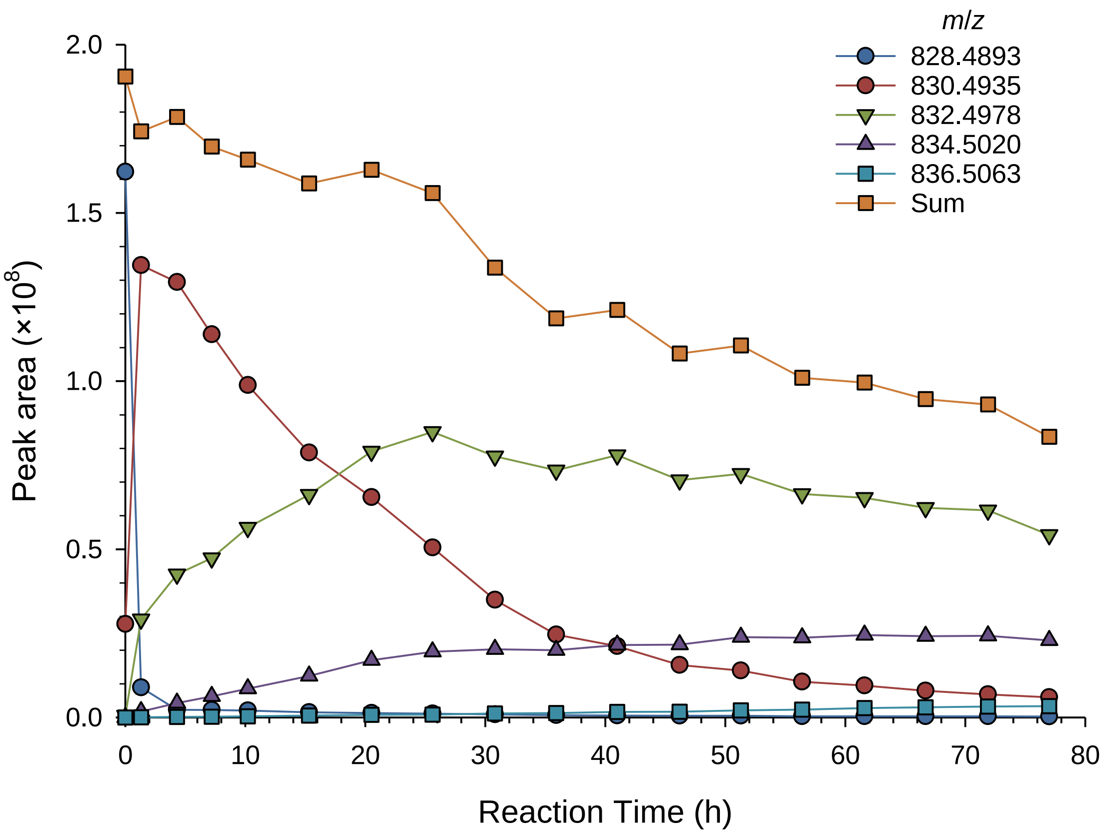

Figure S4. Acid-catalysed incorporation of 0–4 ^{18}O atoms into AZA3 based on analysis of LC–HRMS full-scan spectra for the ions listed (± 5 ppm), with the sum of all peak areas to demonstrate overall losses of AZA3. Note that this analytical approach does not correct for signal interference arising from isotopes naturally present in AZAs, such as ^{13}C.

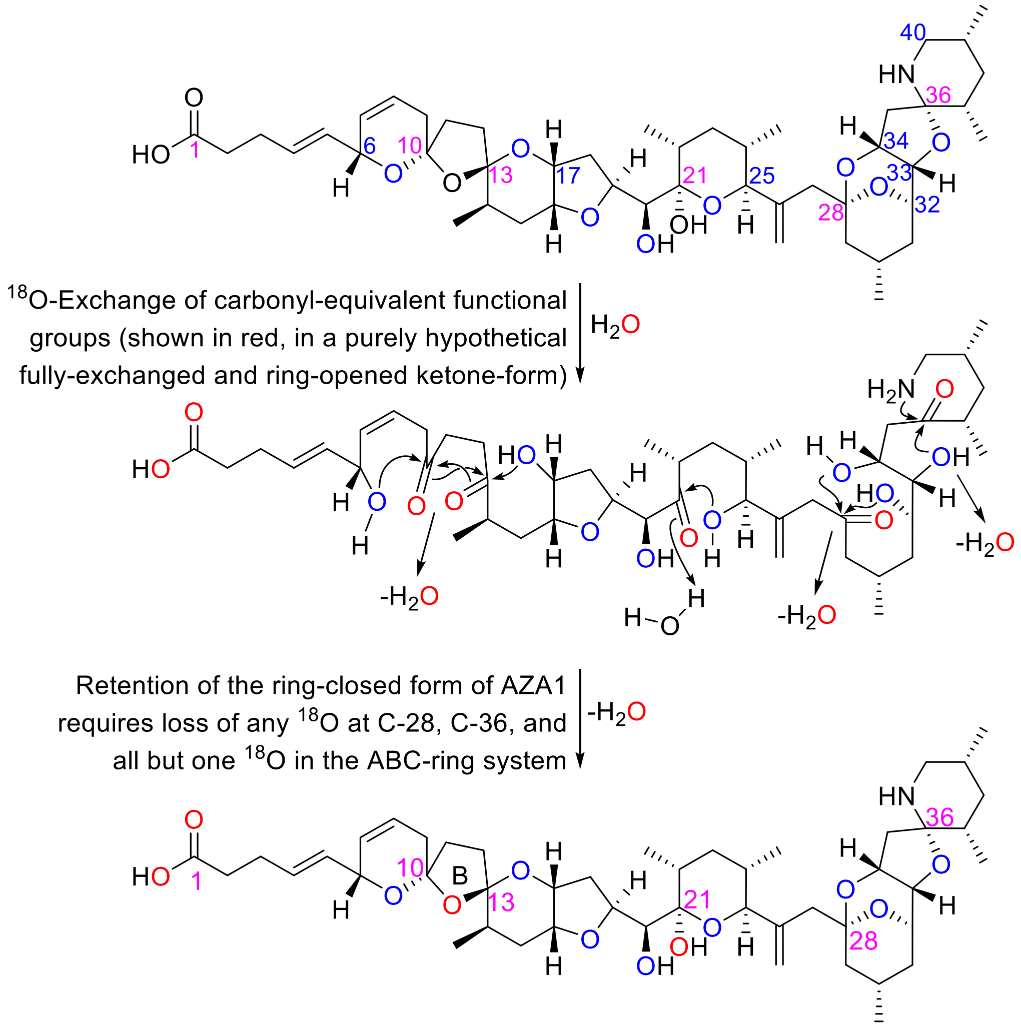

Figure S5. Top, the structure of AZA1 with all non-exchangeable alcohol-derived or ether-oxygens (blue, with blue atom-numbers), and all carbonyl-equivalent carbon atoms indicated with cyan atom-numbers (comprising the carboxylic acid at C-1, ketals at C-10, C-13, and C-28, a hemiketal at C-21, and a hemiaminal ether at C-36); middle, a purely hypothetical illustrative ring-opened structure in which all the carbonyl-equivalent centres have been exchanged with oxygen-labelled (red) water, and; bottom, the same structure but with the ring closures that establish the carboxylic acid, ketal, and hemiaminal ether groups present in the original AZA1 structure. Note that this figure is not intended to represent a mechanism or intermediate in the exchange reactions, but rather to illustrate why incorporation of labelled oxygen in AZA1 is only possible at C-1 (×2), C-21, and in ring-B.

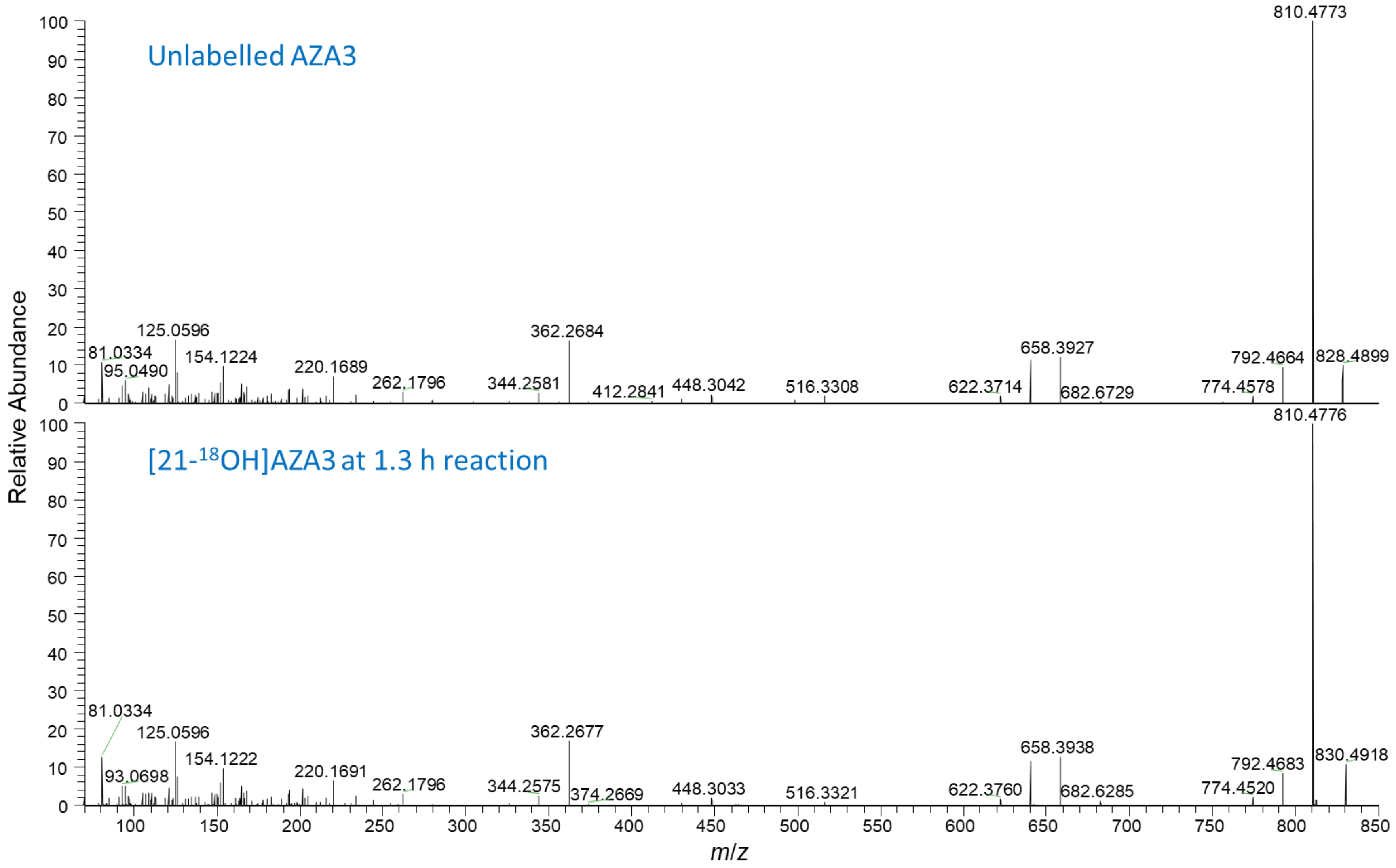

Figure S6. LC–HRMS/MS spectra of: top, AZA3 ($[M+H]^+$ *m/z* 828.5), and; bottom, [21-^{18}OH]AZA3 ($[M+H]^+$ *m/z* 830.5) after 1.3 h of exchange with $H_2{}^{18}O$ in the presence of TFA. Note the absence of ^{18}O in product-ions except for the neutral loss of $H_2{}^{18}O$ from the precursor-ion.

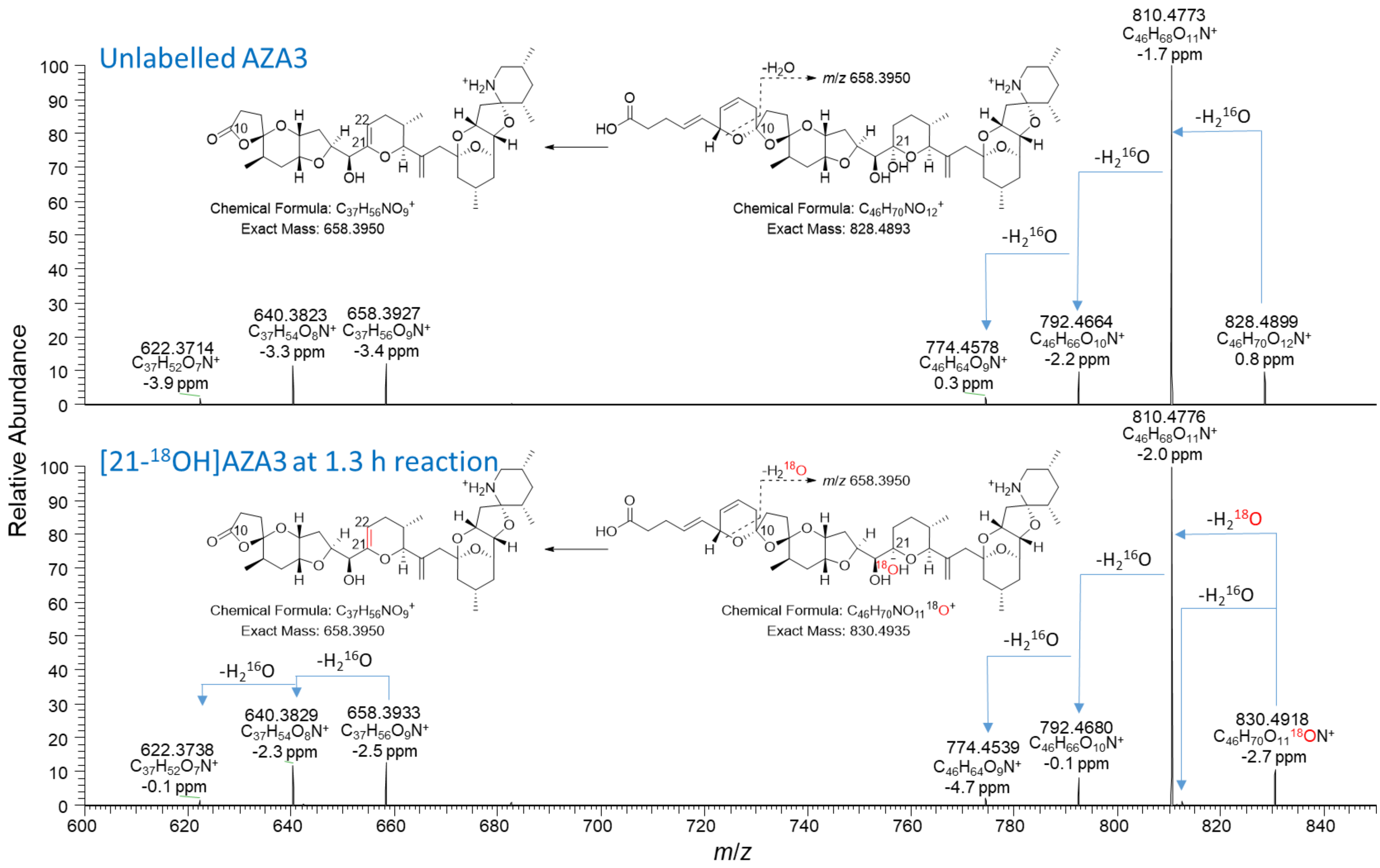

Figure S7. Expansion of the LC–HRMS/MS spectra, from Figure S3, of: top, AZA3 ($[M+H]^+$ *m/z* 828.5), and; bottom, [21-^{18}OH]AZA3 ($[M+H]^+$ *m/z* 830.5) after 1.3 h of exchange with $H_2{}^{18}O$ in the presence of TFA. Note the absence of ^{18}O-label in product-ions from the [21-^{18}OH]AZA3 except for the neutral loss of $H_2{}^{18}O$ from the precursor ion.

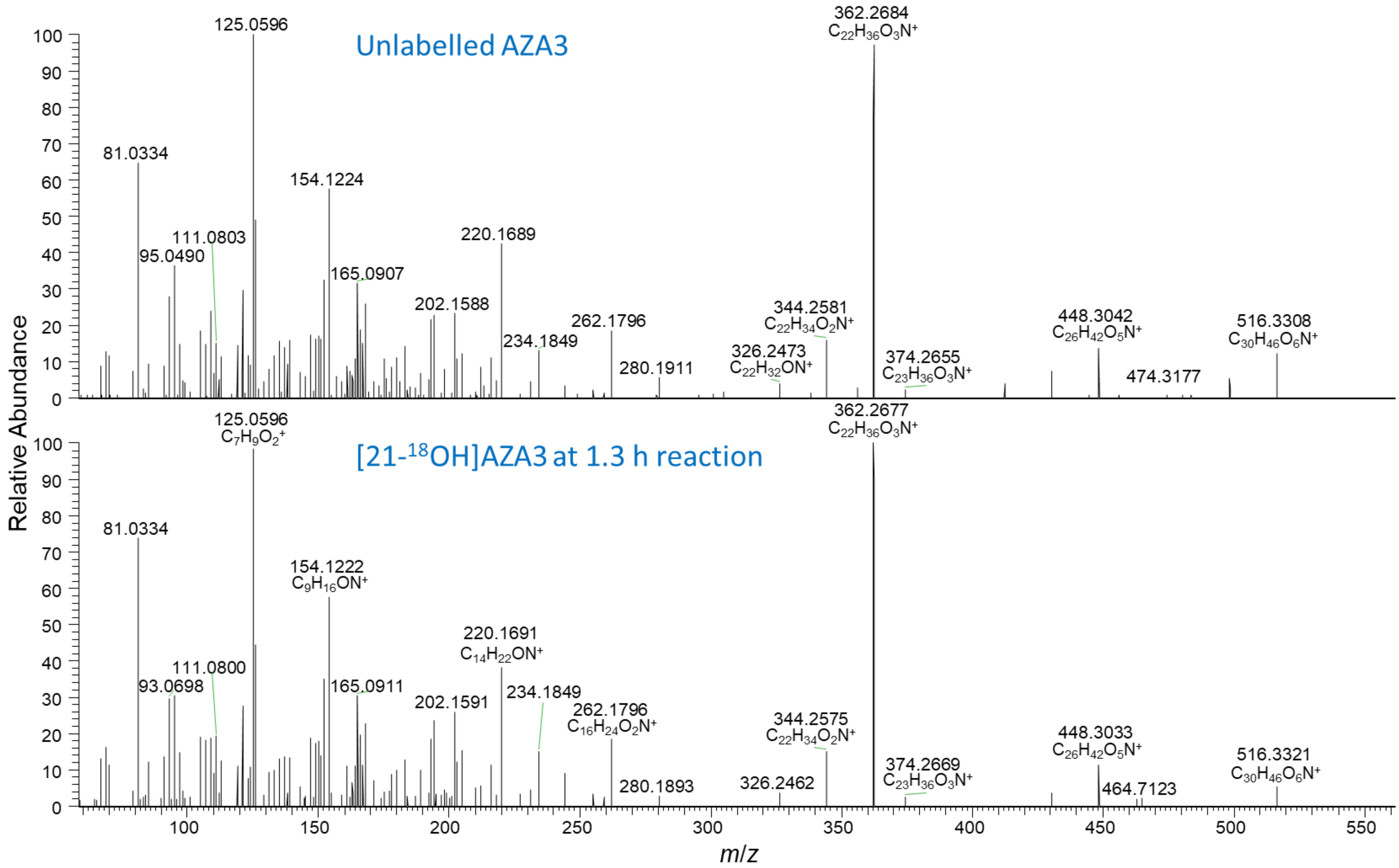

Figure S8. Expansion of the LC–HRMS/MS spectra, from Figure S3, of: top, AZA3 ($[M+H]^+$ *m/z* 828.5), and; bottom, [21-^{18}OH]AZA3 ($[M+H]^+$ *m/z* 830.5) after 1.3 h of exchange with $H_2{}^{18}O$ in the presence of TFA. Note the absence of ^{18}O in product-ions.

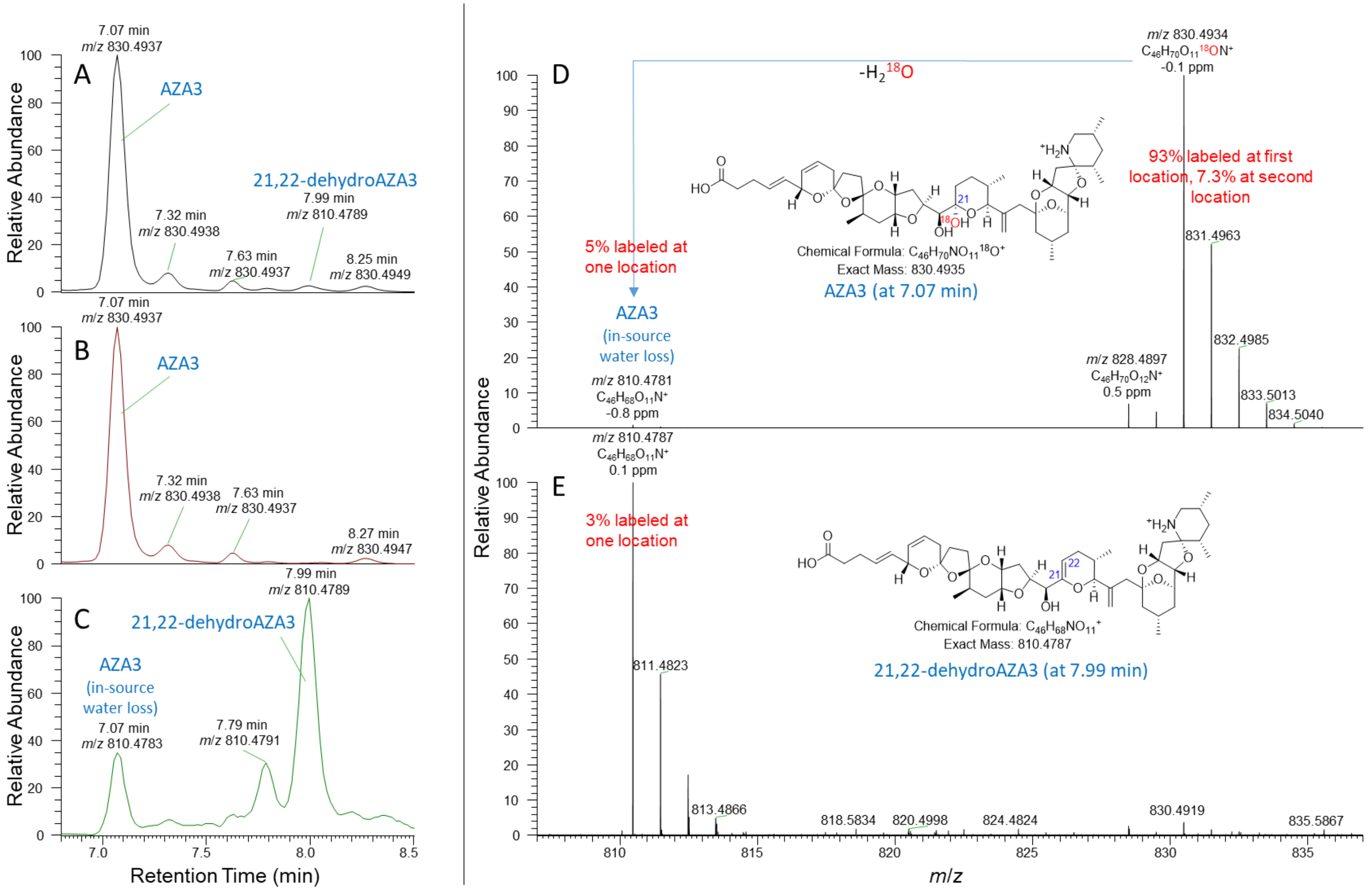

Figure S9. Left, LC–HRMS chromatograms extracted at *m/z* of $[M + H]^+$ of ^{18}O-labeled AZA3 and 21,22-dehydroAZA3 (i.e. AZA25) after 1.3 h of exchange in the kinetic study: A, isotopomers of AZA3 (*m/z* 828.4893 + 830.4935 + 832.4977 + 834.5020) plus 21,22-dehydroAZA3 (*m/z* 810.4787 + 812.4829 + 814.4872); B, isotopomers of ^{18}O-labelled AZA3, and; C, isotopomers of ^{18}O-labelled 21,22-dehydroAZA3 (AZA25). Right, full-scan HRMS of: D, the ^{18}O-labelled AZA3, and; E, the ^{18}O-labelled 21,22-dehydroAZA3. The text in red is the estimated ^{18}O-content based on analysis of the isotopomer profile of that ion cluster (i.e. $[M + H]^+$ or $[M + H – water]^+$) with the NRC Isotopic Enrichment Calculator.

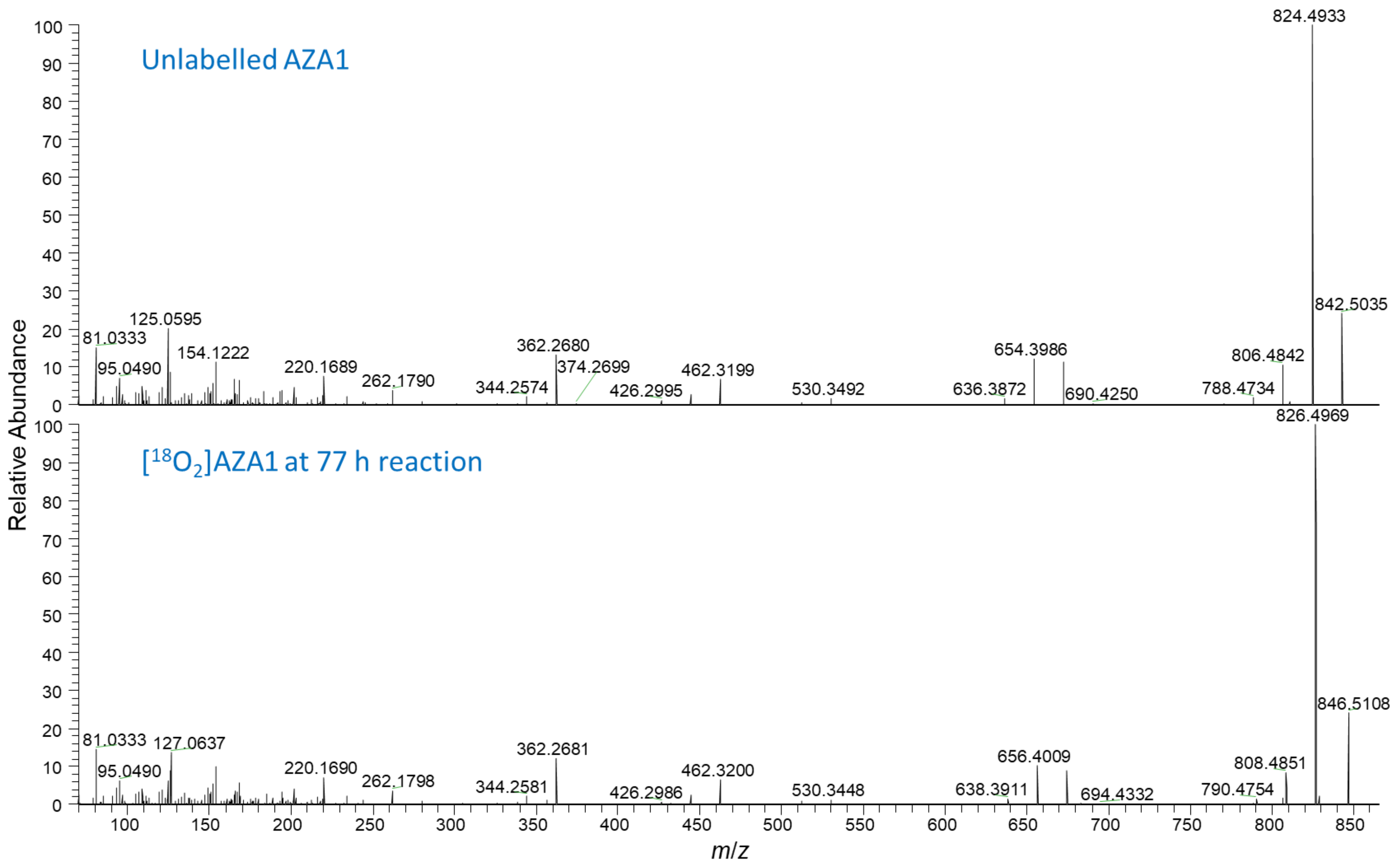

Figure S10. LC–HRMS/MS spectra of: top, AZA1 ([M+H]$^+$ *m/z* 842.5), and; bottom, [$^{18}O_2$]AZA1 ([M+H]$^+$ *m/z* 846.5) after 77 h of exchange with $H_2{}^{18}O$ in the presence of TFA. Note the presence of two ^{18}O atoms only in the precursor-ion, and only one ^{18}O atom in product-ions from the ion cluster associated with retro-Diels–Alder cleavage of the A-ring.

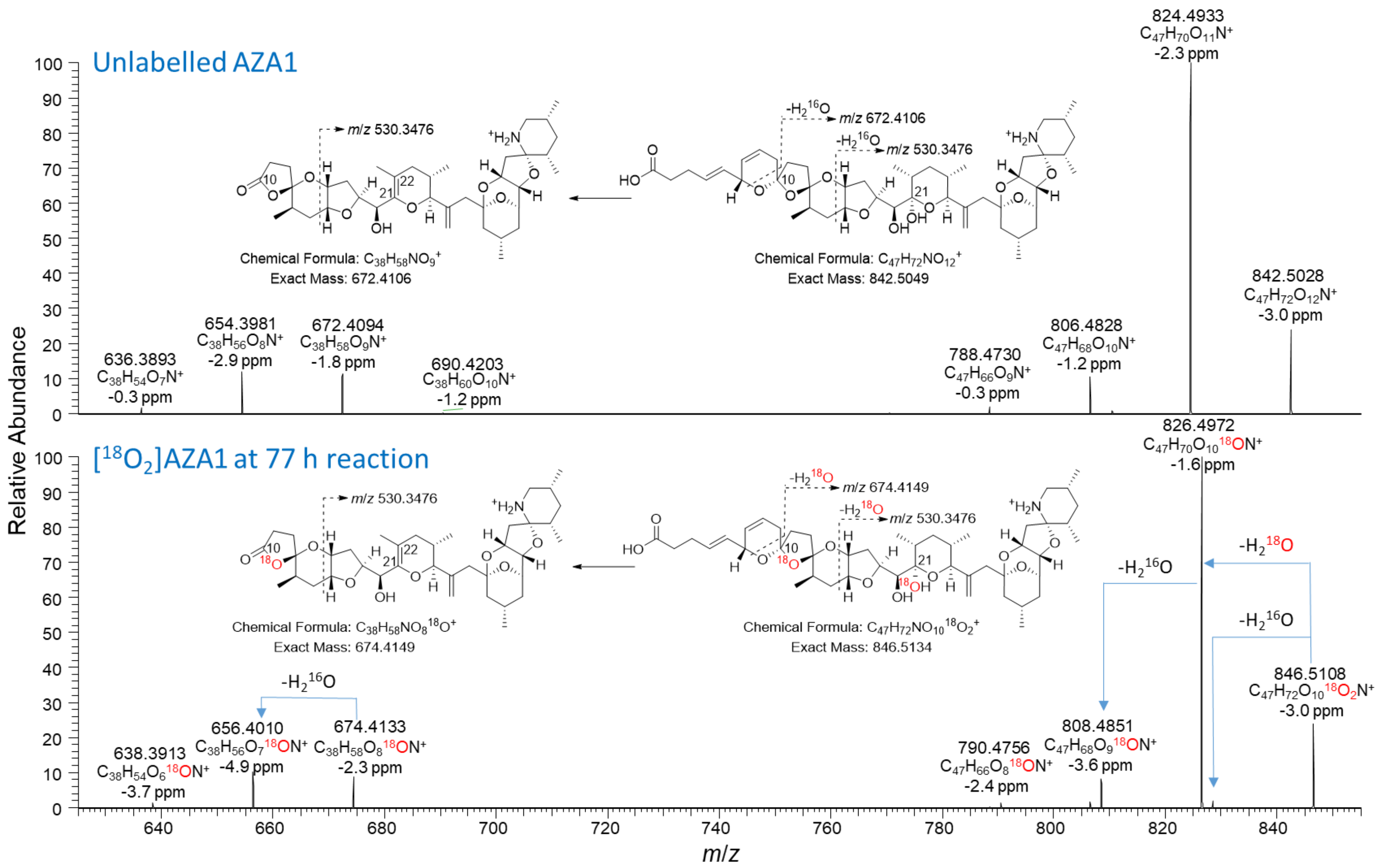

Figure S11. LC–HRMS/MS spectra of: top, AZA1 ($[M+H]^+$ *m/z* 842.5), and; bottom, $[^{18}O_2]$AZA1 ($[M+H]^+$ *m/z* 846.5) after 77 h of exchange with $H_2{}^{18}O$ in the presence of TFA. Note the presence of ^{18}O only in the precursor-ion, its water loss ions, and in product-ions from the ion cluster associated with retro-Diels–Alder cleavage of the A-ring.

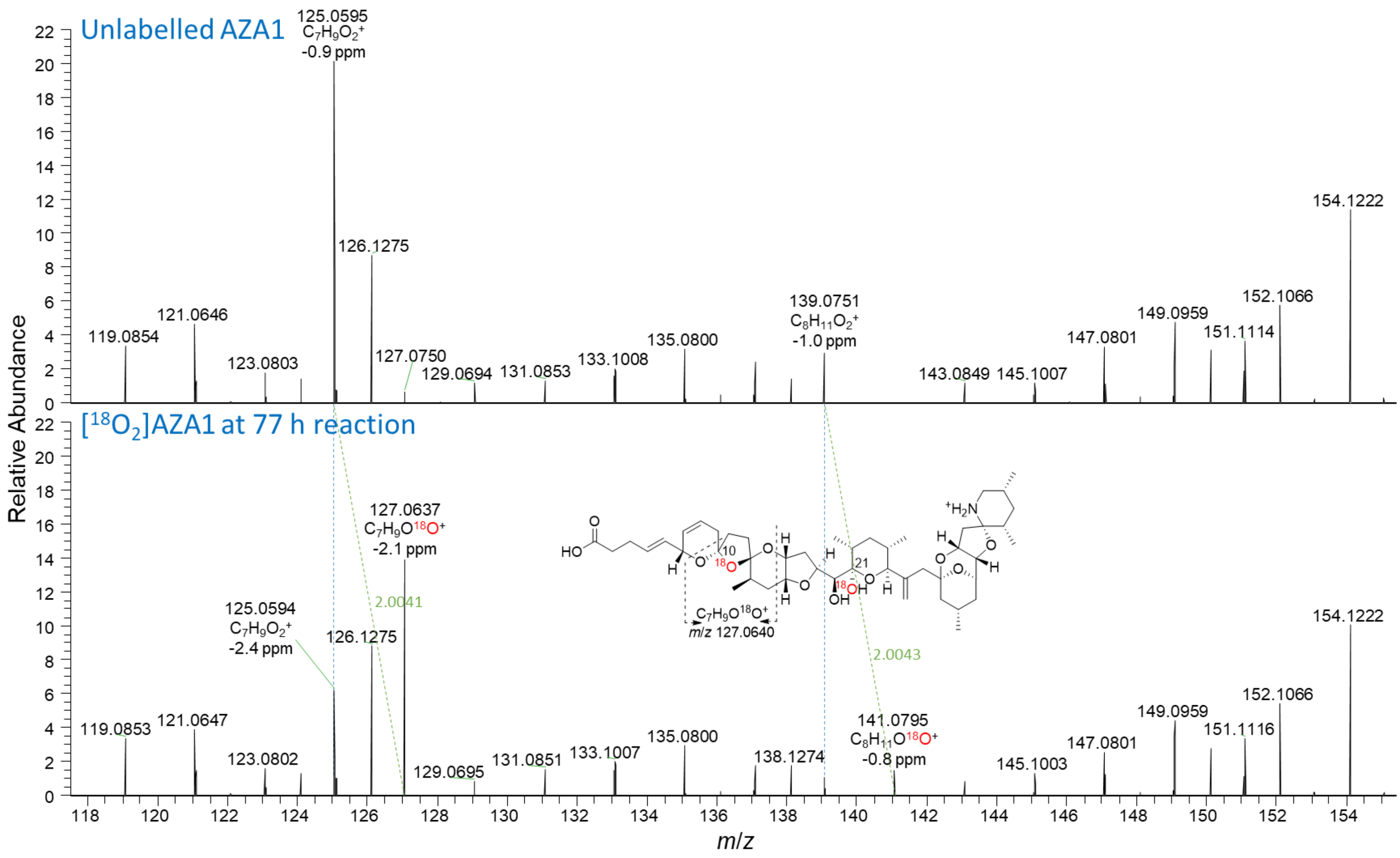

Figure S12. LC–HRMS/MS spectra of: top, AZA1 ([M+H]$^+$ *m/z* 842.5), and; bottom, [$^{18}O_2$]AZA1 ([M+H]$^+$ *m/z* 846.5) after 77 h of exchange with $H_2{}^{18}O$ in the presence of TFA. Note the presence of ^{18}O in the m/z 125.0597, attributable to a fragment containing ring-B of AZA1.

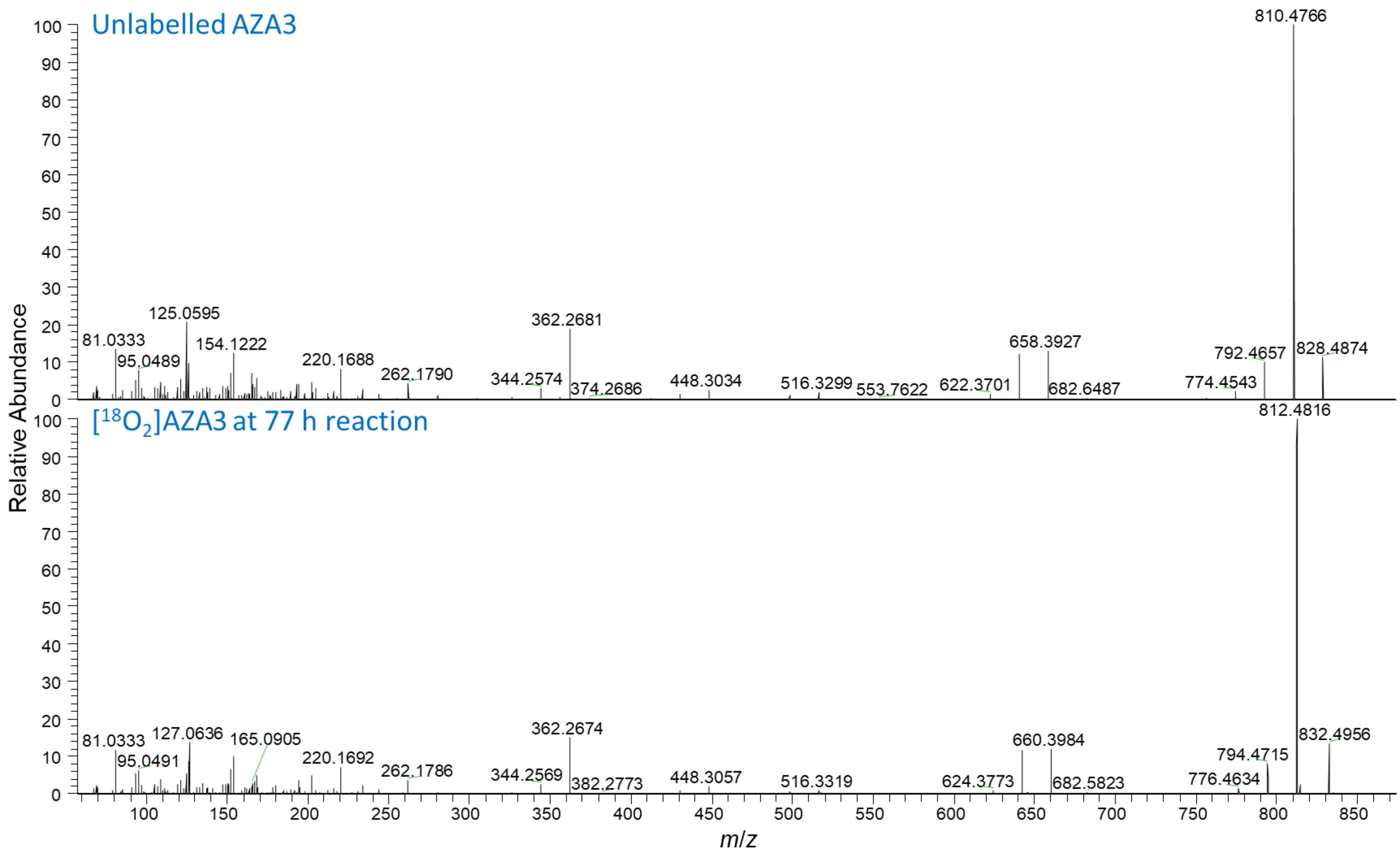

Figure S13. LC–HRMS/MS spectra of: top, AZA3 ($[M+H]^+$ *m/z* 828.5), and; bottom, $[^{18}O_2]$AZA3 ($[M+H]^+$ *m/z* 832.5) after 77 h of exchange with $H_2{}^{18}O$ in the presence of TFA. Note the presence of ^{18}O only in the precursor-ion, its water loss ions, and in product-ions from the ion cluster associated with retro-Diels–Alder cleavage of the A-ring.

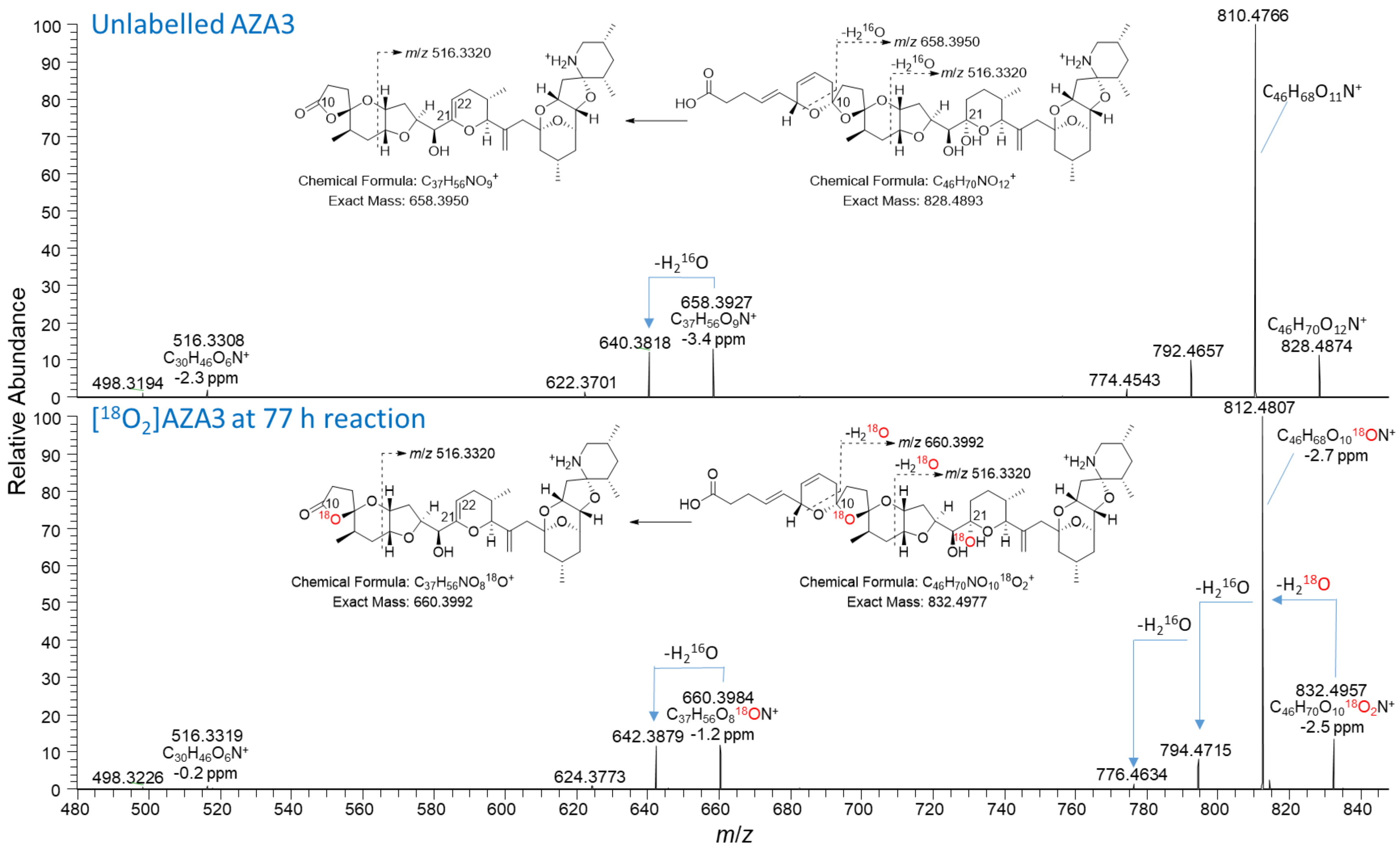

Figure S14. LC–HRMS/MS spectra of: top, AZA3 ([M+H]$^+$ *m/z* 828.5), and; bottom, [$^{18}O_2$]AZA3 ([M+H]$^+$ *m/z* 832.5) after 77 h of exchange with $H_2{}^{18}O$ in the presence of TFA. Note the presence of ^{18}O only in the precursor-ion, its water loss ions, and in product-ions from the ion cluster associated with retro-Diels–Alder cleavage of the A-ring.

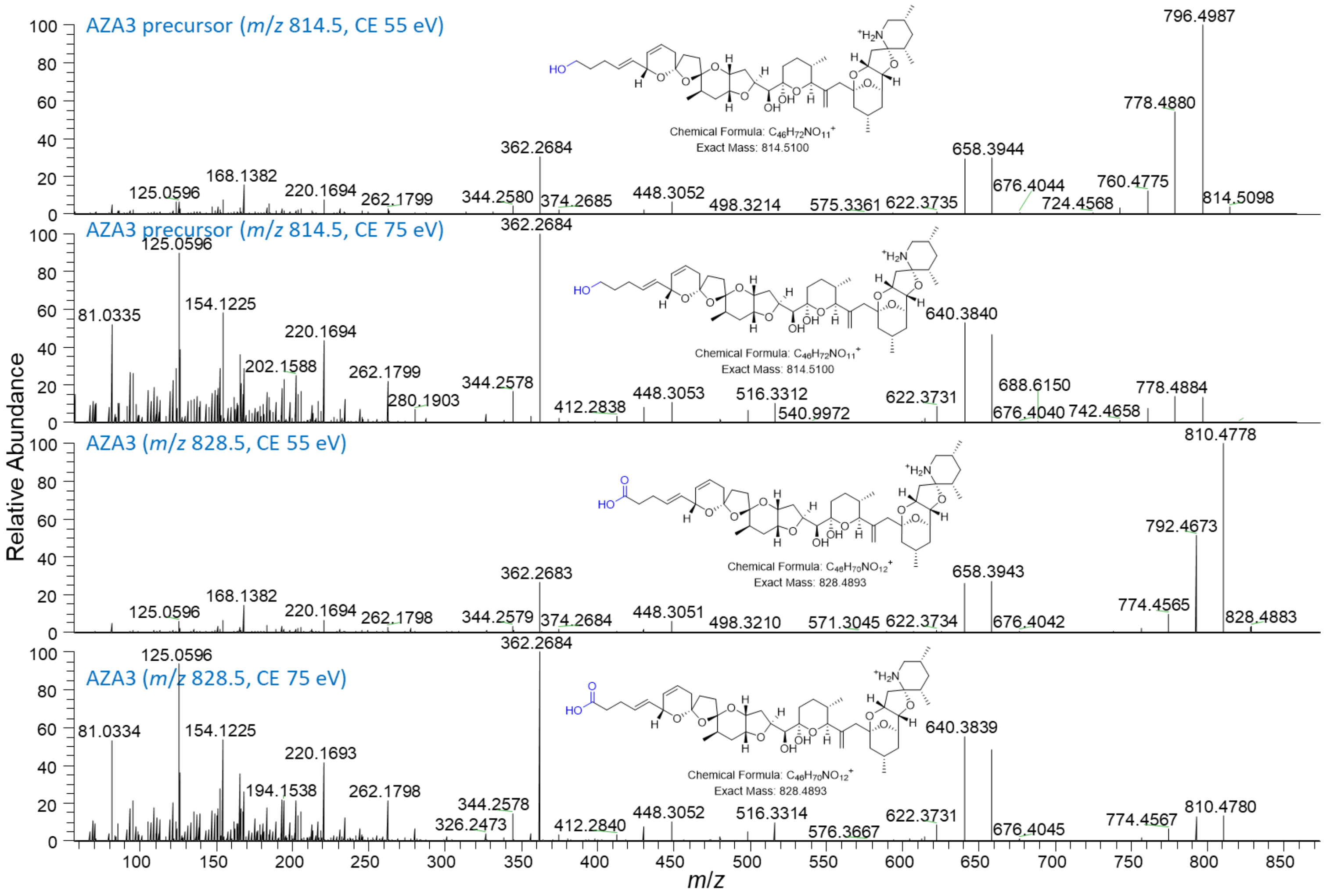

Figure S15. LC–HRMS/MS spectra of synthetic AZA3 (lower panels) and its minor contaminating synthetic C-1 alcohol precursor (upper panels) using two CEs, obtained during previous studies to determine the absolute stereochemistry of AZA3.[1-2] Note the nearly identical relative intensities of corresponding ions from the two compounds when obtained with the same CE, including the product-ion at *m*/*z* 125.0597, which would have *m*/*z* 109.0648 (not observed) in the C-1 alcohol if this ion originated from C-1 to C-7.

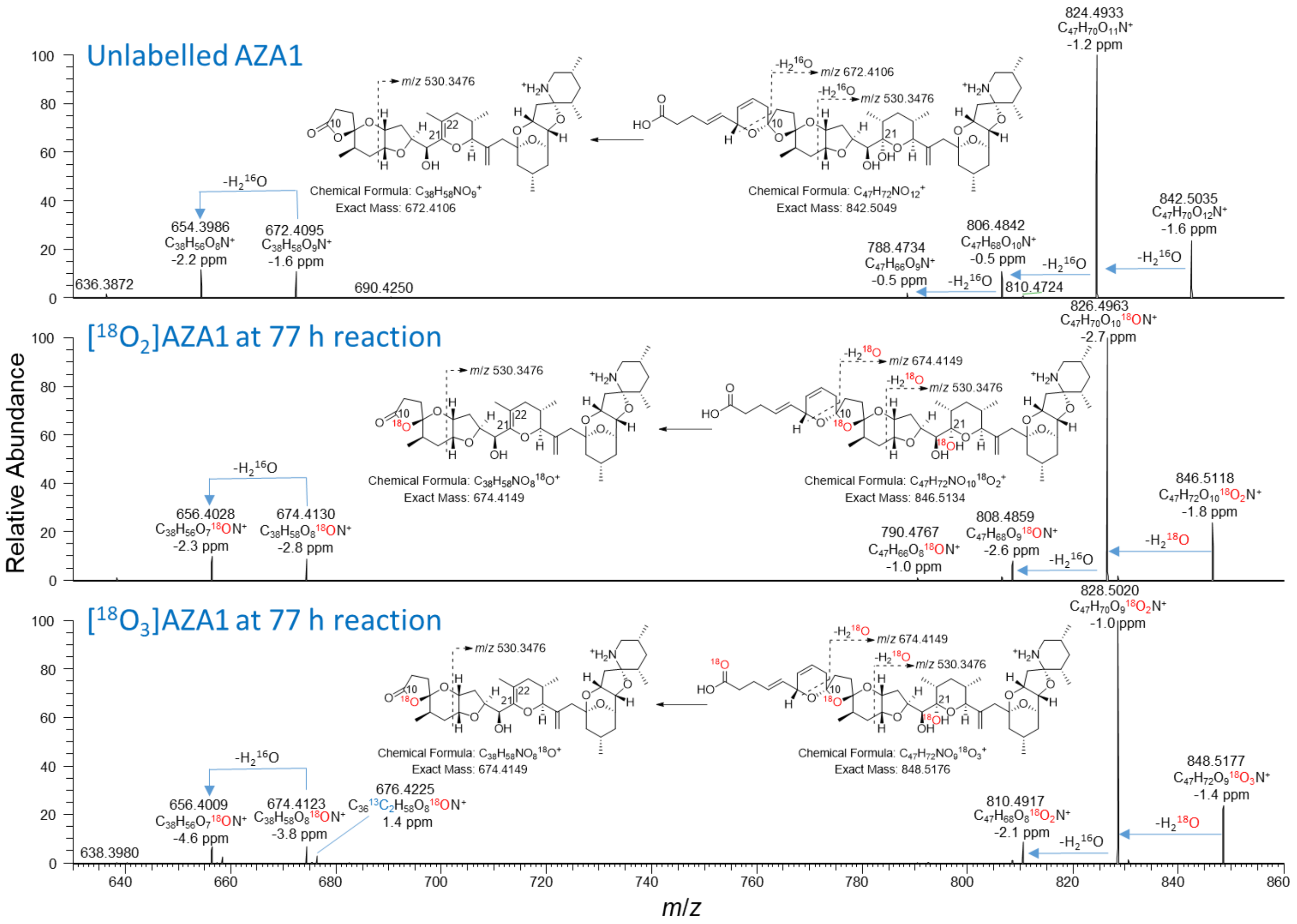

Figure S16. LC–HRMS/MS spectra of: top, AZA1 ($[M+H]^+$ *m/z* 842.5); middle, $[^{18}O_2]$AZA1 ($[M+H]^+$ *m/z* 846.5, and; bottom, $[^{18}O_3]$AZA1 ($[M+H]^+$ *m/z* 848.5) after 77 h of exchange with $H_2{}^{18}O$ in the presence of TFA. Note the presence of three ^{18}O atoms in the precursor-ion but of only one in the product-ions from the ion cluster associated with retro-Diels–Alder cleavage of the A-ring.

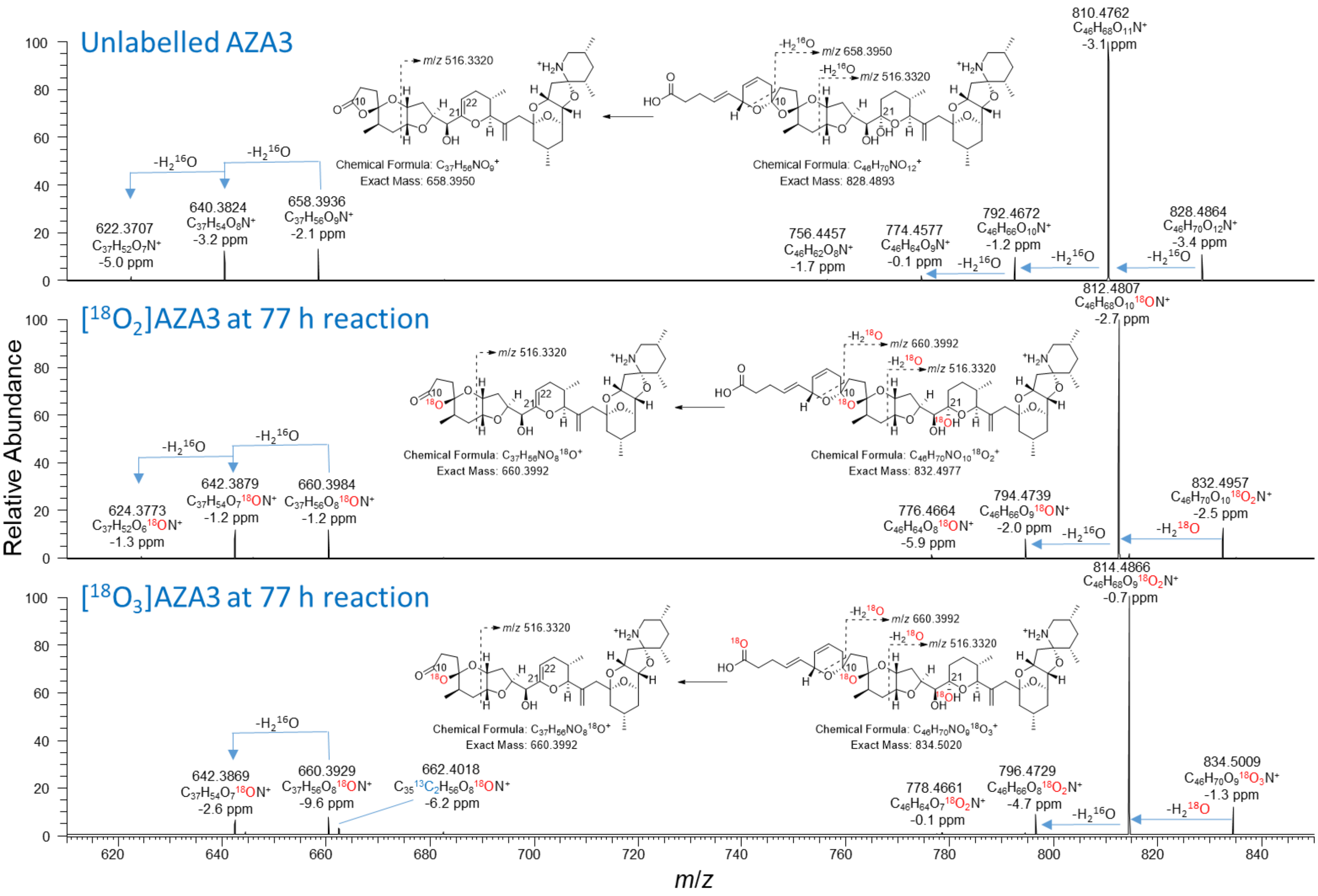

Figure S17. LC–HRMS/MS spectra of: top, AZA3 ($[M+H]^+$ *m/z* 828.5); middle, $[^{18}O_2]$AZA3 ($[M+H]^+$ *m/z* 832.5, and; bottom, $[^{18}O_3]$AZA3 ($[M+H]^+$ *m/z* 834.5) after 77 h of exchange with $H_2{}^{18}O$ in the presence of TFA. Note the presence of three ^{18}O atoms in the precursor-ion but of only one in the product-ions from the ion cluster associated with retro-Diels–Alder cleavage of the A-ring.

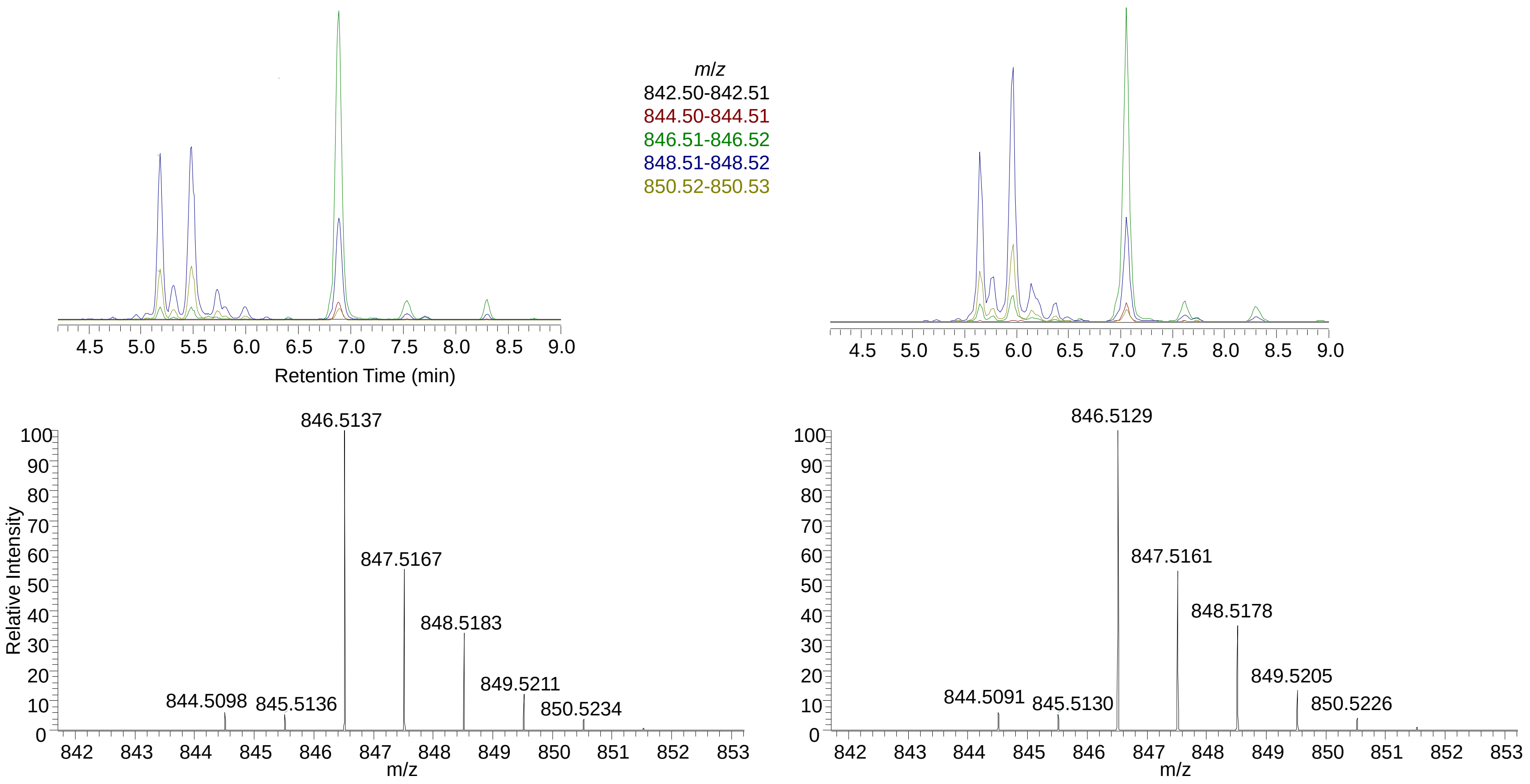

Figure S18. LC–HRMS analysis of ^{18}O-labelled AZA1 spiked into a mixture of methanol and water (left column) re-analyzed after storage at −20 °C for 18 months (right column), showing chromatograms for 0–4 isotope incorporations into the AZA1 structure (top row), and corresponding isotopic profile for the AZA1 peak (bottom row).

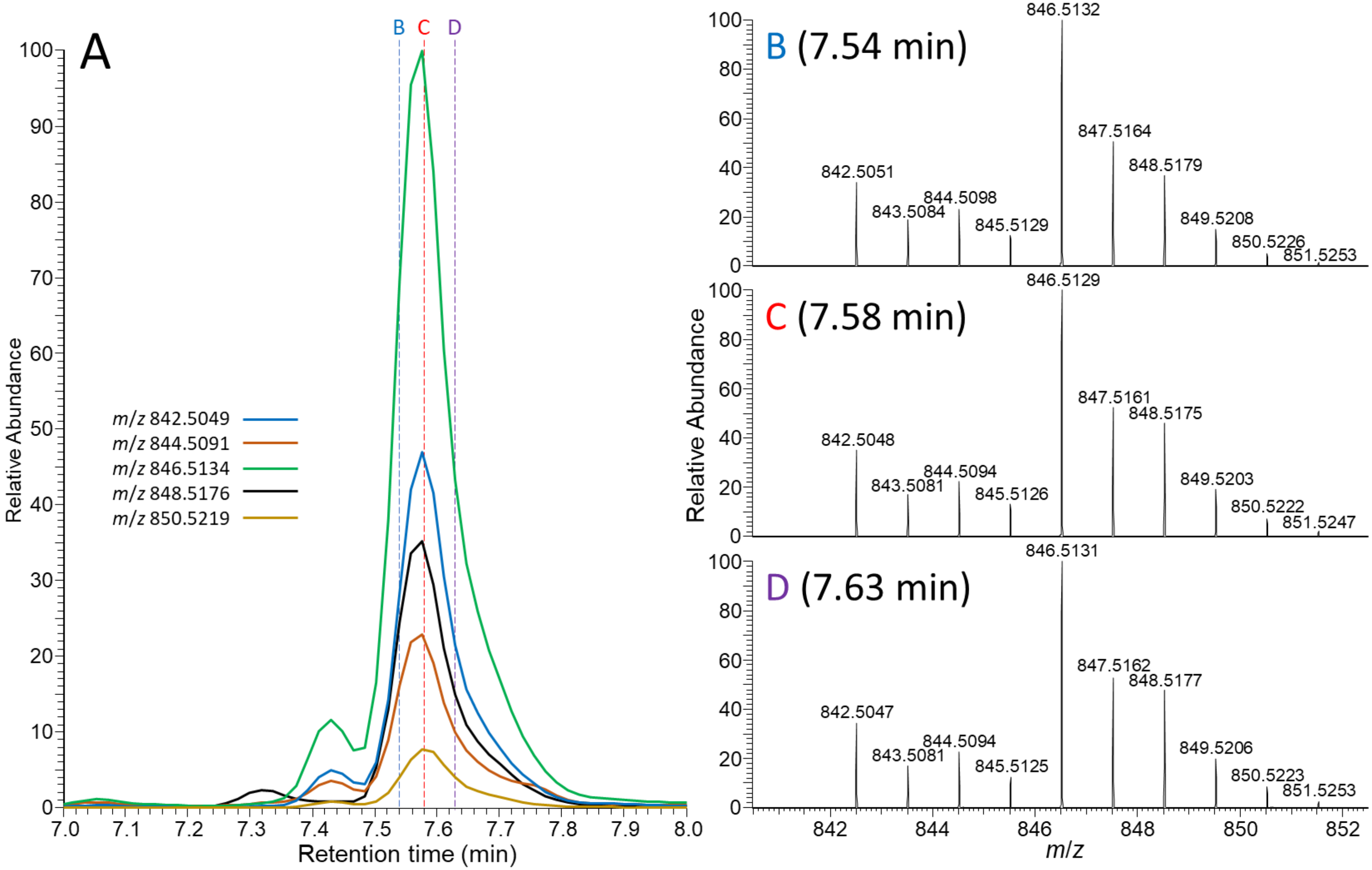

Figure S19. A, Full-scan LC–HRMS chromatogram, extracted at the indicated *m*/*z* values, of an extract of FDMT1 spiked with the labelled AZA1 during the isotope-dilution quantitation study, and; B–D, full-scan mass spectra obtained from the chromatogram at the indicated retention times.

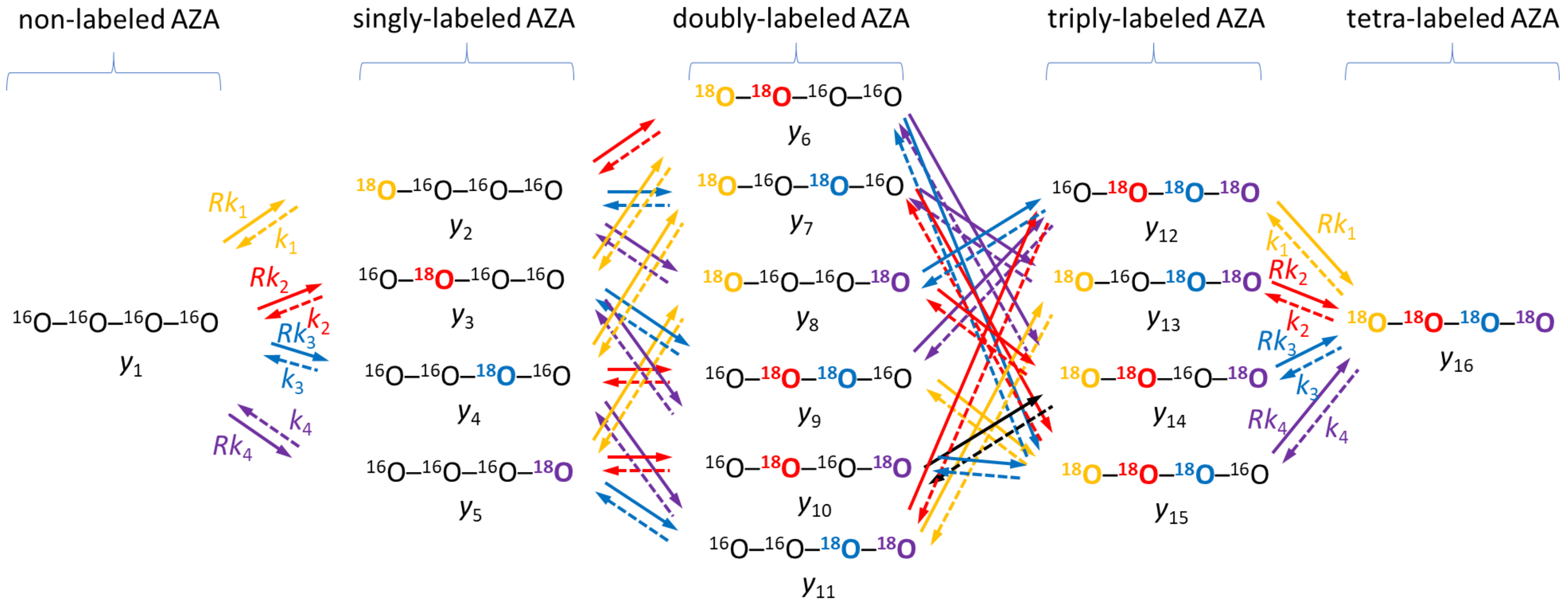

Scheme S1. Schematic showing the 16 possible ^{18}O-isotopologues of AZAs, and the 64 pseudo-first-order reactions (32 forward, and 32 reverse reactions) between them. Rate constants k_1 to k_4 were assigned to exchange at each position (indicated with bold yellow, red, blue or purple), but with the constraint that $k_1 = k_2$ to represent the pool containing the two chemically equivalent exchangeable oxygen atoms (the carboxylic acid at C-1). The rate of the forward reaction, relative to the reverse reaction, is controlled by the coefficient R, which was assumed to have the same value for the exchange reaction at each pool. According to this model, the variable R is the ratio of the of the rate constants for incorporation and loss of ^{18}O at any exchangeable position, and in the absence of significant kinetic isotope effects should represent the ratio of exchangeable ^{18}O to ^{16}O in the reaction solution. The entities y_1 to y_{16} are the amount-fractions of each of the possible isotopologues (so $y_1 + \ldots + y_{16} = 1$), with only 4 additional variables to consider in the model: $k_1 = k_2$, k_3, k_4, and R for each AZA exchange reaction. The mathematical approach to fitting this model to the experimentally measured time-course for the production of the non-, singly-, doubly-, triply, and tetra-^{18}O-labeled AZAs is outlined shown Scheme 2 (additional details can be obtained on request from J. Meija, National Research Council, Ottawa, ON, Canada). This approach is an extension of that recently applied to ^{18}O-incorporation into Caribbean ciguatoxins and gambierones.[3]

Scheme S2. Mathematical analysis of ^{18}O-incorporation data.

The following 16 differential equations describing the changes of each of the 16 isotopologues of the AZA, with entities and constants as defined in Scheme S2. This approach is an extension of that recently applied to ^{18}O-incorporation into Caribbean ciguatoxins and gambierones.[3]

```
# Non-labeled isotopomer
# y1 = oooo

dy1 = + k1*y2 + k2*y3 + k3*y4 + k4*y5 -R*k1*y1 -R*k2*y1 -R*k3*y1 -R*k4*y1

# Mono-labeled isotopomers
# y2 = xooo
# y3 = oxoo
# y4 = ooxo
# y5 = ooox

dy2 = +R*k1*y1 + k2*y6 + k3*y7 + k4*y8 - k1*y2 - R*k2*y2 - R*k3*y2 - R*k4*y2
dy3 = +R*k2*y1 + k1*y6 + k3*y9 + k4*y10 - R*k1*y3 - k2*y3 - R*k3*y3 - R*k4*y3
dy4 = +R*k3*y1 + k1*y7 + k2*y9 + k4*y11 - R*k1*y4 - R*k2*y4 - k3*y4 - R*k4*y4
dy5 = +R*k4*y1 + k1*y8 + k2*y10 + k3*y11 - R*k1*y5 - R*k2*y5 - R*k3*y5 - k4*y5

# Doubly-labeled isotopomers
# y6 = xxoo
# y7 = xoxo
# y8 = xoox
# y9 = oxxo
# y10 = oxox
# y11 = ooxx

dy6 = +R*k1*y3 +R*k2*y2 + k3*y15 + k4*y14 - k1*y6 - k2*y6 - R*k3*y6 - R*k4*y6
dy7 = +R*k1*y4 +R*k3*y2 + k2*y15 + k4*y13 - k1*y7 - R*k2*y7 - k3*y7 - R*k4*y7
dy8 = +R*k1*y5 +R*k4*y2 + k3*y13 + k2*y14 - k1*y8 - R*k2*y8 - R*k3*y8 - k4*y8
dy9 = +R*k2*y4 +R*k3*y3 + k4*y12 + k1*y15 - R*k1*y9 - k2*y9 - k3*y9 - R*k4*y9
dy10 = +R*k2*y5 +R*k4*y3 + k1*y14 + k3*y12 - R*k1*y10 - k2*y10 - R*k3*y10 - k4*y10
dy11 = +R*k3*y5 +R*k4*y4 + k1*y13 + k2*y12 - R*k1*y11 - R*k2*y11 - k3*y11 - k4*y11

# Triply-labeled isotopomers
# y12 = oxxx
# y13 = xoxx
# y14 = xxox
# y15 = xxxo

dy12 = +R*k4*y9 + R*k3*y10 + R*k2*y11 + k1*y16 - R*k1*y12 - k2*y12 - k3*y12 - k4*y12
dy13 = +R*k1*y11 + R*k3*y8 + R*k4*y7  + k2*y16 - k1*y13 - R*k2*y13 - k3*y13 - k4*y13
dy14 = +R*k1*y10 + R*k2*y8 +R*k4*y6 + k3*y16 - k1*y14 - k2*y14 - R*k3*y14 - k4*y14
dy15 = +R*k1*y9 + R*k2*y7 + R*k3*y6 + k4*y16 - k1*y15 - k2*y15 - k3*y15 - R*k4*y15
```

Scheme S2 (cont.). Mathematical analysis of ^{18}O-incorporation data.

Tetra-labeled isotopomer
y16 = xxxx

dy16 = +R*k1*y12 + R*k2*y13 + R*k3*y14 + R*k4*y15 - k1*y16 - k2*y16 - k3*y16 - k4*y16

The results obtained from fitting the above model to the experimental isotopic composition data, using maximum likelihood, are shown below (for alternative graphical representations, see also Figures 3–6):

	AZA1		AZA3	
Quantity*	Value	Uncertainty	Value	Uncertainty
ln (k_1) = ln (k_2)	−9.65	0.08	−9.66	0.13
ln (k_3)	−6.56	0.09	−6.59	0.17
ln (k_4)	−5.65	0.28	−2.58	0.21
R	28.9	1.5	29.6	2.7

*Pseudo-first-order rate constants k_1–k_4 in h^{-1}.

The uncertainties of the model parameters were evaluated using non-parametric bootstrap method by performing the MLE fitting to the resampled data set whereby one or two observations were omitted from the dataset. The standard deviation of the resulting set of (N=25) rate constants was taken as the estimate of their uncertainty. Note that the value of R agrees with the expected value in 97% $H_2{}^{18}O$ medium, that is, 0.97/(1 – 0.97) = 32.

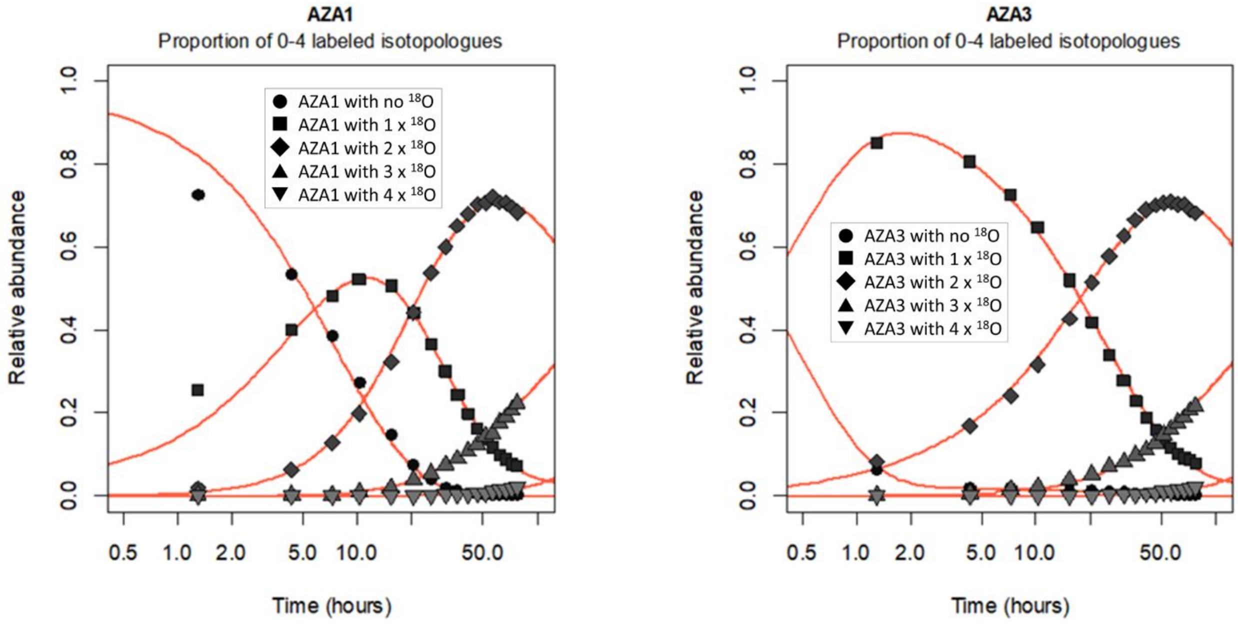

Additional details can be obtained on request from J. Meija, National Research Council, Ottawa, ON, Canada.

Table S1. Measured concentrations of AZA1 and AZA3 (µg/g) for the in-house reference material RM-AZA-Mus, from the Quantitation study. LC–MS-based values are mean results (uncertainty) using external calibration, or using IDMS with singly-, doubly- or triply-^{18}O-labelled AZA peaks from the spiked isotopically-labelled stock solution.[a]

		RM-AZA-Mus	
Method	Calibration	AZA1	AZA3
LC–HRMS	External Calibrant	0.54 (0.04)	0.016 (0.007)
	IDMS 1×^{18}O	0.54 (0.03)	0.011 (0.002)
	IDMS 2×^{18}O	0.54 (0.02)	0.008 (0.007)
	IDMS 3×^{18}O	0.55 (0.03)	0.011 (0.002)
LC–MS/MS	External Calibrant	0.51 (0.02)	0.013 (0.001)
	IDMS 1×^{18}O	0.58 (0.03)	0.010 (0.002)
	IDMS 2×^{18}O	0.56 (0.03)	0.009 (0.002)
	IDMS 3×^{18}O	0.55 (0.03)	0.012 (0.003)
Reported value (uncertainty)[a]		**0.44 (0.04)**	**0.005 (0.002)**

[a]Non-certified value and stated uncertainty for RM-AZA-Mus.[4] AZA concentrations are reported as the sums of each AZA plus its 37-epimer. See Table 1 for corresponding results for CRM-FDMT1 and CRM-AZA-Mus. Uncertainties given for experimental results consider the contributions from calibrant uncertainty, calibration model, replicate analysis, and sample preparation.

Literature Cited

1. Kenton, N. T.; Adu-Ampratwum, D.; Okumu, A. A.; McCarron, P.; Kilcoyne, J.; Rise, F.; Wilkins, A. L.; Miles, C. O.; Forsyth, C. J., Stereochemical definition of the natural product (6*R*,10*R*,13*R*,14*R*,16*R*,17*R*,19*S*,20*S*,21*R*,24*S*,25*S*,28*S*,30*S*,32*R*,33*R*,34*R*,36*S*,37*S*,39*R*)-azaspiracid-3 via total synthesis and comparative analyses. *Angew. Chem., Int. Ed.* **2018,** *57* (3), 810–813.

2. Kenton, N. T.; Adu-Ampratwum, D.; Okumu, A. A.; Zhang, Z.; Chen, Y.; Nguyen, S.; Xu, J.; Ding, Y.; McCarron, P.; Kilcoyne, J.; Rise, F.; Wilkins, A. L.; Miles, C. O.; Forsyth, C. J., Total synthesis of (6*R*,10*R*,13*R*,14*R*,16*R*,17*R*,19*S*,20*R*,21*R*,24*S*,25*S*,28*S*,30*S*,32*R*,33*R*,34*R*,36*S*,37*S*,39*R*)-azaspiracid-3 reveals non-identity with the natural product. *Angew. Chem., Int. Ed.* **2018,** *57* (3), 805–809.

3. Mudge, E. M.; Meija, J.; Uhlig, S.; Robertson, A.; McCarron, P.; Miles, C. O., Production and stability of oxygen-18 labeled Caribbean ciguatoxins and gambierones. *Toxicon* **2022,** *211*, 11–20.

4. Quilliam, M. A.; Reeves, K.; MacKinnon, S. L.; Craft, C.; Whyte, H.; Walter, J. A.; Stobo, L.; Gallacher, S., Preparation of reference materials for azaspiracids. In *Molluscan Shellfish Safety. Proceedings of the 5th International Conference on Molluscan Shellfish Safety, Galway, Ireland, June 14th–18th, 2004*, Henshilwood, K.; Deegan, B.; McMahon, T.; Cusack, C.; Keaveney, S.; Silke, J.; O' Cinneide, M.; Lyons, D.; Hess, P., Eds. The Marine Institute, Rinville, Oranmore, Galway, Ireland: Galway, Ireland, 2006; pp 111−115.
